# The E3 Ubiquitin Ligase PRAJA1: A Key Regulator of Synaptic Dynamics and Memory Processes with Implications for Alzheimer’s Disease

**DOI:** 10.3390/ijms26072909

**Published:** 2025-03-23

**Authors:** Chuhan Li, Yan Yan, Oliver Stork, Ruling Shen, Thomas Behnisch

**Affiliations:** 1 State Key Laboratory of Medical Neurobiology, Institutes of Brain Science, MOE Frontiers Center for Brain Science, Fudan University, Shanghai 200032, China; 2 Department of Genetics and Molecular Neurobiology, Institute of Biology, Otto-von-Guericke University Magdeburg, 39120 Magdeburg, Germany; oliver.stork@ovgu.de; 3 Shanghai Laboratory Animal Research Center, Shanghai 201203, China

**Keywords:** proteasome-ubiquitin system, *Pja1*, hippocampus, synaptic plasticity, learning and memory, spinophilin, Alzheimer’s disease

## Abstract

The precise regulation of synaptic function by targeted protein degradation is fundamental to learning and memory, yet the roles of many brain-enriched E3 ubiquitin ligases in this process remain elusive. Here, we uncover a critical and previously unappreciated role for the E3 ubiquitin ligase PRAJA1 in orchestrating synaptic plasticity and hippocampus-dependent memory. Utilizing C57BL/6 and 5xFAD male mice and employing a multi-faceted approach including protein biochemistry, molecular biology, in vitro electrophysiology, and behavioral assays, we demonstrate that long-term potentiation (LTP) induction triggers a rapid, proteasome-dependent downregulation of PRAJA1 within the CA1 region of the hippocampus. Critically, selective knockdown of PRAJA1 in vivo profoundly enhanced both object recognition and spatial memory, while disrupting normal exploratory behavior. Mechanistically, we reveal that PRAJA1 acts as a key regulator of synaptic architecture and transmission: its downregulation leads to a reduction in key synaptic proteins and spine density, influencing the excitatory/inhibitory balance and facilitating synaptic plasticity. Conversely, increased PRAJA1 expression potentiates GABAergic transmission. Furthermore, we identify spinophilin as a novel substrate of PRAJA1, suggesting a direct molecular link between PRAJA1 and synaptic remodeling. Strikingly, our findings implicate dysregulation of PRAJA1 in the pathogenesis of Alzheimer’s disease, positioning PRAJA1 as a potential therapeutic target for cognitive enhancement in neurodegenerative conditions. These results unveil PRAJA1 as a critical molecular brake on synaptic plasticity and memory formation, offering a promising new avenue for understanding and potentially treating memory impairment.

## 1. Introduction

The precise regulation of synaptic plasticity, the cellular foundation of learning and memory, critically depends on the ubiquitin-proteasome system (UPS), a sophisticated machinery that orchestrates targeted protein degradation [1]. Within this system, E3 ubiquitin ligases hold a pivotal position, conferring substrate specificity to the ubiquitination process and thereby precisely controlling the protein landscape at the synapse. Given their fundamental role, E3 ligases represent compelling targets for strategies aimed at enhancing cognitive function. Moreover, dysregulation of the ubiquitin-proteasome system, and in particular RING finger E3 ubiquitin ligases, is increasingly recognized as an important factor in human neurological disease. Illustrative examples include mutations in *ARIH2* associated with neurodevelopmental disorders such as autism and intellectual disability [2], and the role of *HOIP* in accelerating neurodegeneration in spinobulbar muscular atrophy (SBMA) [3]. Furthermore, beyond neurodegenerative contexts, *TRIM32*, among other RING finger ligases, is critical for neuronal development, influencing dendrite arborization [4] and alpha-synuclein levels, with implications for Parkinson’s disease [5]. Collectively, these findings underscore the broad and critical involvement of RING finger ligases in neuronal health and their potential as therapeutic targets in a range of neurological diseases. Among the numerous E3 ubiquitin ligases expressed in the brain, PRAJA1, an E3 RING-H2 ligase initially identified for its role in liver development in mice [6] has garnered increasing attention for its diverse functions and potential involvement in cognitive processes. Beyond its general role in cellular homeostasis [6,7,8], accumulating evidence suggests that PRAJA1 plays a multifaceted role within the nervous system. This is highlighted by its diverse substrates, which include NRAGE (a p75 neurotrophin receptor-interacting protein) [9], enhancer of zeste homolog 2 (EZH2) [10], and DLXIN-1 [11], pointing to its involvement in the development of the skeletal, muscular, and nervous systems [10,11,12], suggesting broader neurodevelopmental and potentially cognitive functions.

For instance, PRAJA1 interacts with the homology domain of NRAGE, facilitating PRAJA1-mediated ubiquitination and subsequent proteasomal degradation of NRAGE [9,11]. This interaction modulates Msx2 and Dlx5-dependent transcription [9,11], and has been shown to negatively regulate neurite outgrowth [9]. Furthermore, PRAJA1 is induced during NGF-dependent differentiation [13], suggesting a role in the consolidation of neuritogenesis. The discovery that PRAJA1 mediates the effects of the astrocytic membrane estrogen receptor G protein-coupled receptor 30 (GPR30) on learning and memory by binding to Serpina3 [14] provides a compelling link between astrocytic signaling, PRAJA1 expression, and cognitive function, even suggesting a potential therapeutic avenue for cognitive decline in peri- and postmenopausal women due to GPR30 positively regulating PRAJA1 expression through the CREB signaling pathway [14]. Adding to its complexity, PRAJA1 interacts with and inhibits the accumulation of various polyQ proteins associated with neurodegenerative diseases, such as ataxin-3, huntingtin, and TDP-43 [15,16], suggesting a protective role against proteinopathy. In contrast to its neuroprotective roles, emerging evidence also implicates PRAJA1 in neurodevelopmental processes. Indeed, links to neurodevelopmental disorders have surfaced from findings that *PRAJA1* gene deletions or mutations are observed in patients with craniofrontonasal syndrome, trigonocephaly, or epilepsy [17,18]. Furthermore, supporting the crucial role of PRAJA1 in normal brain function, mice with a targeted *Pja1* knock-in mutation (p.Arg365Cys, equivalent to p.Arg376Cys in humans) exhibit a significant reduction in PRAJA1 protein levels and display increased susceptibility to seizures induced by pentylenetetrazole [18], underscoring its importance for proper neurological development and function.

Within the hippocampus, a brain region critical for memory formation, PRAJA1 is prominently expressed in pyramidal neurons and dentate granule cells [19], positioning it as a potential regulator of synaptic plasticity underlying memory. While these diverse lines of evidence suggest important roles for PRAJA1 in neuronal function and behavior, the precise contribution of PRAJA1 to activity-dependent synaptic plasticity within the hippocampus, and its consequent impact on memory formation, remains largely unexplored. Intriguingly, studies have reported a downregulation of PRAJA1 in Alzheimer’s disease (AD) models [20], raising the critical question of whether this dysregulation contributes to the synaptic and cognitive impairments characteristic of AD.

Therefore, this study directly addresses this critical gap by investigating the role of PRAJA1 in regulating synaptic plasticity and hippocampus-dependent memory. We hypothesized that PRAJA1 acts as a key modulator of synaptic function, and its dysregulation contributes to memory impairments, particularly in the context of Alzheimer’s disease. To test this, we examined the impact of altered PRAJA1 expression on synaptic transmission, neuronal excitability, and memory-related behaviors in vivo, utilizing both wild-type mice and the 5×FAD mouse model of Alzheimer’s disease. Our findings reveal that PRAJA1 is a dynamically regulated E3 ligase that acts as a critical molecular brake on synaptic plasticity and memory formation in the hippocampus. We further identify spinophilin as a novel PRAJA1 substrate and provide evidence implicating PRAJA1 dysregulation in the synaptic deficits observed in Alzheimer’s disease, highlighting its potential as a therapeutic target for cognitive enhancement.

## 2. Results

### 2.1. PRAJA1 Expression and Activity-Dependent Regulation in the Hippocampus

**Figure 1:**
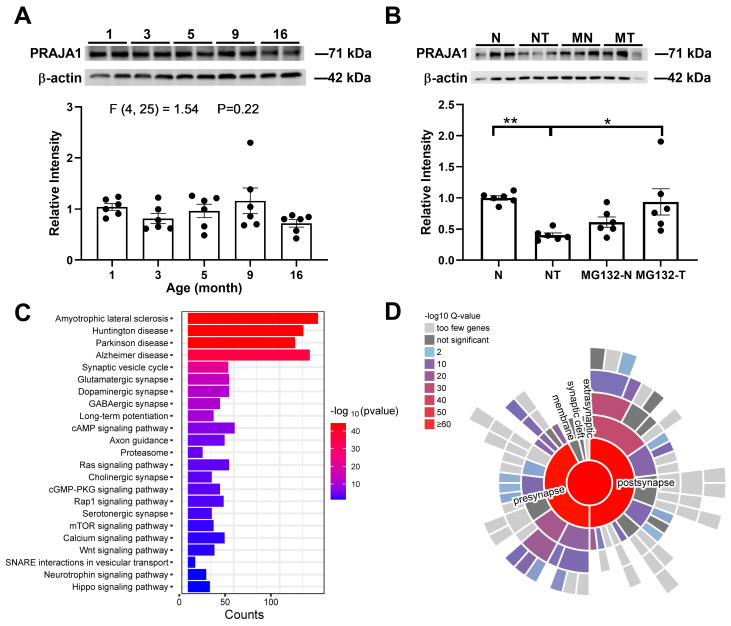
PRAJA1 Expression and Activity-Dependent Degradation in the Hippocampus. (**A**) Quantification of PRAJA1 protein levels in the CA1 region across different ages of mice, showing stable expression. Representative Western blots are shown above (*n* = 6 per group; One-way ANOVA, *p* > 0.05). (**B**) Activity-dependent decrease in PRAJA1 protein following LTP induction. High-frequency stimulation (STET) significantly reduced PRAJA1 levels, an effect prevented by the proteasome inhibitor MG132. Representative Western blots are shown above. (*n* = 6 per condition; One-way ANOVA: Sidak’s test: * *p* = 0.0097, ** *p* = 0.024; N: no drug, normal conditions; NT: no drug, but STET; MG-132-N (MN): MG-132; MG-132-T (MT): MG-132 and STET). (**C**) KEGG pathway enrichment analysis of PRAJA1-interacting proteins. The top 23 enriched pathways, ranked by *p*-value, demonstrate significant associations with neurodegenerative diseases and synaptic plasticity. Bar length indicates pathway richness, and color intensity represents significance (−log_10_ (*p*-value)). (**D**) Sunburst plot illustrating cellular component enrichment analysis of PRAJA1-interacting proteins. The size of each segment reflects the number of associated proteins, with color intensity indicating enrichment significance. A large proportion of interacting proteins are localized to synaptic components.

To gain deeper insights into the involvement of *Pja1* in neurobiological processes, we investigated the age-dependence of endogenous PRAJA1 expression in the hippocampal formation and assessed changes in PRAJA1 levels following the induction of synaptic plasticity in the CA1 region. We found that endogenous PRAJA1 protein levels in the adult CA1 region remained stable across a 16-month period, indicating consistent basal expression (Figure 1A). To investigate the link between PRAJA1 and synaptic plasticity, we induced long-term potentiation (LTP) in CA1 hippocampal slices. High-frequency stimulation (STET) triggered a rapid and significant reduction in PRAJA1 protein levels within 50 min (Figure 1B). This activity-dependent decrease was blocked by the proteasome inhibitor MG132, demonstrating that LTP induces PRAJA1 degradation via the ubiquitin-proteasome system (Figure 1B).

To identify potential functions, we characterized PRAJA1-interacting proteins in the CA1 region using co-immunoprecipitation and mass spectrometry. Enrichment analysis revealed that these interacting proteins are significantly associated with pathways implicated in neurodegenerative diseases and synaptic plasticity, particularly those related to LTP and both excitatory and inhibitory synapses (Figure 1C). Furthermore, a significant proportion (591 out of 1697) of these interacting proteins are annotated as synaptic genes in the SynGO database [21], highlighting a strong link between PRAJA1 and synaptic function (Figure 1D). These data also suggest that PRAJA1 in CA1 is associated with several neurodegenerative diseases, including amyotrophic lateral sclerosis, Huntington’s disease, Parkinson’s disease, and Alzheimer’s disease.

### 2.2. PRAJA1 Deficiency in CA1 Disrupts Synaptic Structure and Function

**Figure 2:**
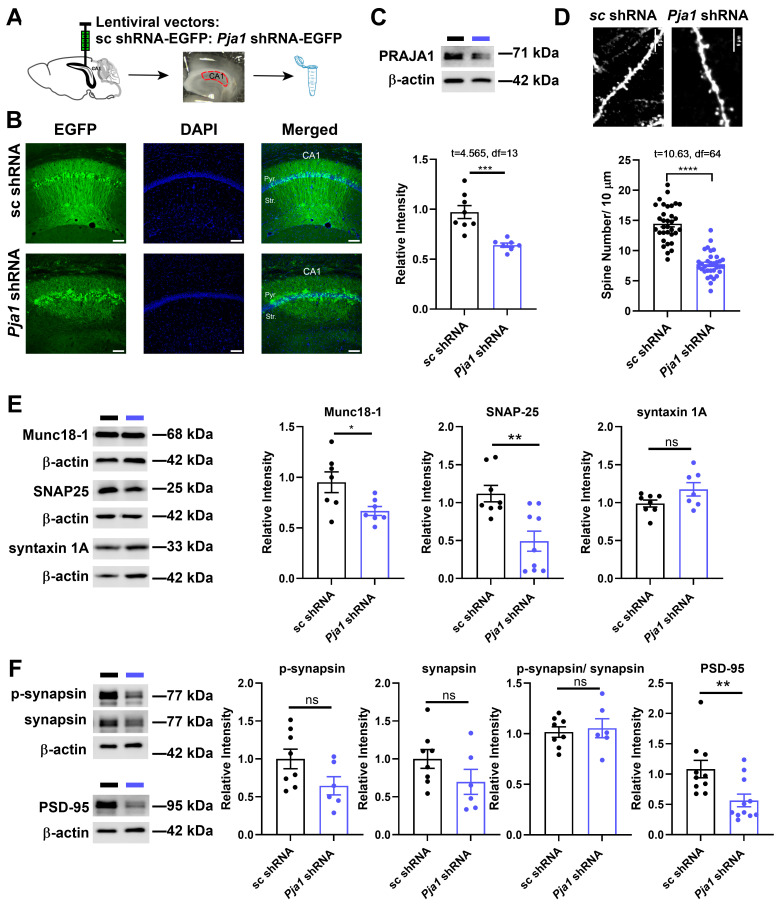
*Pja1* Knockdown in CA1 Reduces Spine Density and Alters Synaptic Protein Expression. (**A**) Schematic of bilateral lentiviral injection targeting the CA1 region. (**B**) Representative immunofluorescence images of the CA1 region showing GFP expression (green), DAPI nuclear staining (blue), and merged images of mice injected with either scrambled shRNA (sc shRNA, top row) or *Pja1* shRNA (bottom row) are shown. Scale bar: 100 μm. (**C**) Quantification of PRAJA1 protein levels following lentiviral injection, showing a significant reduction in the *Pja1* shRNA group. (*n* = 8 sc shRNA, *n* = 7 *Pja1* shRNA, unpaired *t*-test, *** *p* = 0.0005). (**D**) Representative images of dendritic spines (top) and quantification of spine density (bottom), demonstrating a significant decrease in *Pja1* knockdown mice. Scale bar: 5 μm. (*n* = 33 spines from 3 mice per group; unpaired *t*-test; **** *p* < 0.0001). (**E**) Representative Western blots and quantification of pre-synaptic protein levels, showing significant reductions in Munc18-1 and SNAP-25 in the *Pja1* shRNA group. (Munc18-1: *n* = 7 per group, * *p* = 0.025; SNAP-25: sc shRNA: *n* = 7; *Pja1* shRNA: *n* = 9, *p* > 0.05, ** *p* = 0.006; syntaxin 1A: sc shRNA: *n* = 8; *Pja1* shRNA: *n* = 7, *p* > 0.05 ns, unpaired *t*-test). (**F**) Representative Western blots and quantification of post-synaptic protein levels, revealing a significant reduction in PSD-95 in the *Pja1* shRNA group (Synapsin (total and phosphorylated): sc shRNA: *n* = 8; *Pja1* shRNA: *n* = 7, *p* > 0.05 ns; PSD-95: sc shRNA: *n* = 10; *Pja1* shRNA: *n* = 11, *p* > 0.05, ** *p* = 0.008 unpaired *t*-test.

Given the activity-dependent regulation of PRAJA1, we investigated its role in synaptic function by knocking down its expression in the CA1 region using lentiviral delivery of *Pja1* shRNA (Figure 2A,B). This resulted in a significant ~30% reduction in PRAJA1 protein levels compared to control mice receiving scrambled shRNA (Figure 2C; sc shRNA: 0.97 ± 0.065; *Pja1* shRNA: 0.64 ± 0.021). Consistent with a role in synaptic maintenance, *Pja1* knockdown led to a marked decrease in dendritic spine density (Figure 2D; **** *p* < 0.0001). Furthermore, immunoblotting revealed significant reductions in the levels of key pre-synaptic proteins Munc18-1, SNAP-25, and syntaxin 1A in *Pja1*-deficient CA1 (Figure 2E). Post-synaptically, PSD-95 levels were also significantly reduced (Figure 2F), while total and phosphorylated synapsin levels remained unchanged. (Figure 2E).

### 2.3. PRAJA1 Deficiency in CA1 Region Disrupts Glutamatergic and GABAergic Synaptic Transmission, Leading to Enhanced LTP

**Figure 3:**
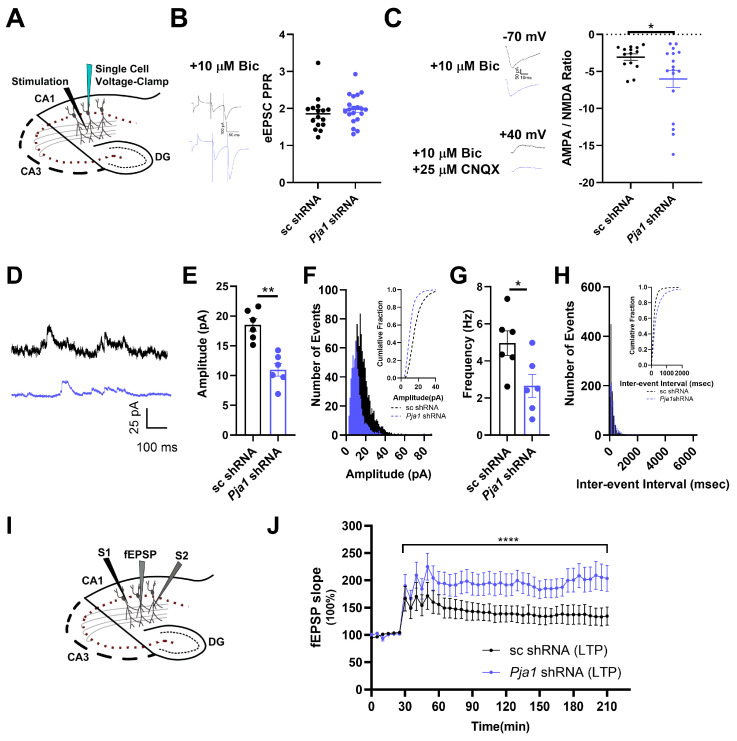
PRAJA1 Knockdown Differentially Alters Glutamatergic and GABAergic Transmission and Enhances LTP. (**A**) Schematic illustrating hippocampal slice preparation and electrode placement for evoked excitatory postsynaptic current (eEPSC) recordings in CA1. (**B**) Representative traces of AMPAR-mediated eEPSCs and quantification of the paired-pulse ratio (PPR). No significant difference was observed between the control (sc shRNA: *n* = 16 cells) and *Pja1* knockdown (*Pja1* shRNA: *n* = 20 cells) groups (*p* > 0.05, unpaired *t*-test). (**C**) Representative traces of eEPSCs at holding potentials of −70 mV and +40 mV to isolate AMPAR and NMDAR currents, respectively. Quantification of the NMDA/AMPA ratio reveals a significant decrease in *Pja1* knockdown neurons compared to controls (sc shRNA: *n* = 15 cells; *Pja1* shRNA: *n* = 19 cells; * *p* < 0.05, unpaired *t*-test). (**D**) Representative traces of miniature inhibitory postsynaptic current (mIPSC) recordings (black line: sc shRNA; blue line: *Pja1* shRNA). (**E**) Quantification of mIPSC amplitude demonstrates a significant reduction in the *Pja1* shRNA group (sc shRNA: *n* = 6; *Pja1* shRNA: *n* = 7; ** *p* < 0.01, unpaired *t*-test). (**F**) Amplitude distribution histograms of mIPSCs, with insets showing cumulative probability plots. Recordings were performed at +40 mV. (**G**) Quantification of mIPSC frequency reveals a significant decrease in the *Pja1* shRNA group (sc shRNA: *n* = 6; *Pja1* shRNA: *n* = 7; * *p* < 0.01, unpaired *t*-test). (**H**) Inter-event interval distribution histograms of mIPSCs. (**I**) Schematic depicting the LTP recording setup. (**J**) Summary of field potential recordings during LTP, showing enhanced potentiation in *Pja1* knockdown slices (sc shRNA: *n* = 7; *Pja1* shRNA: *n* = 6; **** *p* < 0.0001, mixed-effects analysis, Sidak’s test).

To dissect the role of PRAJA1 in synaptic function within the CA1 region, we first examined glutamatergic transmission in CA1 pyramidal neurons using whole-cell recordings. Crucially, while presynaptic release probability, assessed by the paired-pulse ratio (PPR) of AMPAR-mediated evoked EPSCs (eEPSCs), remained unchanged in *Pja1* knockdown neurons (Figure 3A,B; *Pja1* shRNA: 1.98 ± 0.09, *n* = 20 cells; sc shRNA: 1.86 ± 0.12, *n* = 16 cells; *p* > 0.05), postsynaptic function was significantly altered. Specifically, the ratio of NMDAR-mediated to AMPAR-mediated eEPSCs (NMDA/AMPA ratio) was markedly reduced in *Pja1* deficient neurons (Figure 3C; *Pja1* shRNA: 0.227 ± 0.047, *n* = 19 cells; sc shRNA: 0.382 ± 0.051, *n* = 15 cells; *p* < 0.05). Consistent with this functional impairment, quantitative RT-qPCR analysis revealed significant downregulation of mRNA transcripts for key AMPAR (*Gria1*, *Gria2*) and NMDAR (*Grin2a*, *Grin2b*) subunits in the *Pja1* knockdown group (Figure S1A).

We next investigated the impact of PRAJA1 deficiency on GABAergic inhibitory transmission by recording miniature inhibitory postsynaptic currents (mIPSCs). *Pja1* knockdown robustly decreased both the amplitude (Figure 3D,E; *p* < 0.01) and frequency (Figure 3D,G; *p* < 0.01) of mIPSCs, indicating a significant impairment in basal inhibitory synaptic transmission. Furthermore, while the PPR of evoked IPSCs (eIPSCs) was unaffected by *Pja1* knockdown (Figure S1F), the amplitude of eIPSCs was significantly reduced (Figure S1C), suggesting a postsynaptic mechanism underlies this deficit in evoked inhibitory transmission. Supporting this, mRNA levels of key GABA_A_ receptor subunits, *Gabra1* (α1 subunit) and *Gabrb2* (β2 subunit), were significantly reduced in the *Pja1* knockdown group (Figure S2B).

Given these disruptions in both excitatory and inhibitory synaptic transmission, we assessed synaptic plasticity by examining LTP at Schaffer collateral synapses in the CA1 region. Remarkably, despite the observed deficits in basal transmission in single-cell recordings, *Pja1* knockdown slices exhibited a significantly enhanced LTP response following high-frequency stimulation (Figure 3I,J; sc shRNA: *n* = 7; *Pja1* shRNA: *n* = 6; **** *p* < 0.0001). This indicates that while PRAJA1 deficiency impairs basal synaptic transmission, it paradoxically facilitates the induction of synaptic plasticity.

### 2.4. PRAJA1 Regulates Neuronal Excitability and Voltage-Gated Channel Expression

**Figure 4:**
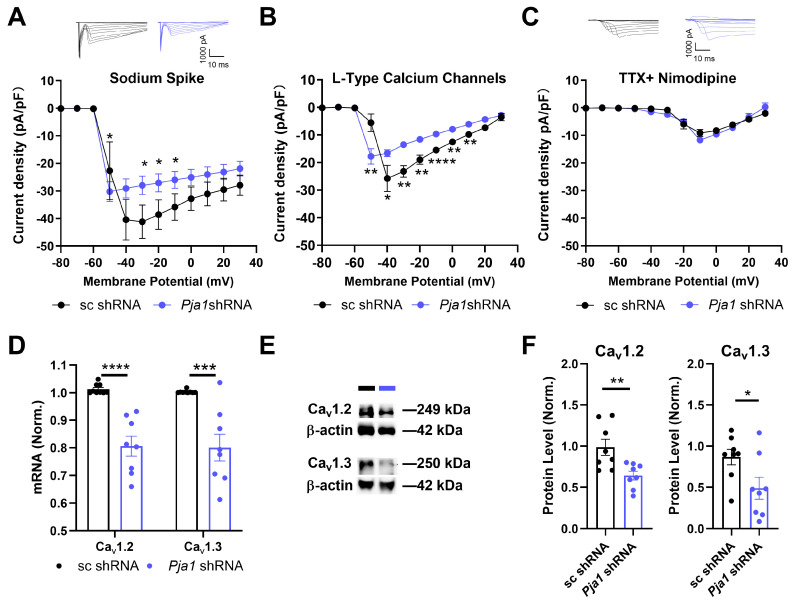
*Pja1* Knockdown Reduces Voltage-Gated Sodium and Calcium Currents and Downregulates Cav1.2 and Cav1.3 Expression. (**A**) Representative current traces and I–V relationship for voltage-gated sodium currents (VGSCs), showing a significant reduction in the *Pja1* shRNA group. (sc shRNA: *n* = 12; *Pja1* shRNA: *n* = 9; * *p* < 0.05). (**B**) I–V relationship for L-type voltage-gated calcium currents (VGCCs), showing a significant reduction in the *Pja1* shRNA group. (sc shRNA: *n* = 12; *Pja1* shRNA: *n* = 9; * *p* < 0.05. ** *p* < 0.01, **** *p* < 0.0001, Mann-Whitney test). (**C**) Representative current traces and I–V relationship for T-type VGCCs, showing no significant difference between groups. (sc shRNA: *n* = 13; *Pja1* shRNA: *n* = 14, *p* > 0.05, Mann-Whitney test). (**D**) Relative mRNA levels of Cav1.2 and Cav1.3, demonstrating significant downregulation in the *Pja1* shRNA group (sc shRNA: *n* = 9; *Pja1* shRNA: *n* = 8; *Cav*1.2: **** *p* < 0.0001; *Cav*1.3: *** *p* < 0.001, unpaired *t*-test). (**E**) Representative Western blots showing reduced Cav1.2 and Cav1.3 protein levels in the *Pja1* shRNA group. (**F**) Quantification of Cav1.2 and Cav1.3 protein levels, confirming significant downregulation in the *Pja1* shRNA group (*n* = 8 per group; Cav1.2: ** *p* < 0.01; Cav1.3: * *p* < 0.05, unpaired *t*-test).

To further investigate the functional consequences of *Pja1* deficiency, we examined neuronal excitability using whole-cell voltage-clamp recordings. *Pja1* knockdown significantly reduced both voltage-gated sodium currents (VGSCs) and L-type voltage-gated calcium currents (VGCCs) in CA1 neurons (Figure 4A–C). Specifically, I_Na_ was significantly lower between −30 and −10 mV, and I_Ca_ was significantly reduced between −50 mV and +10 mV (Figure 4B,C; * *p* < 0.05). In contrast, T-type calcium currents were unaffected by *Pja1* knockdown (Figure 4C). Consistent with the reduction in L-type currents, RT-qPCR and Western blot analysis revealed significant decreases in the mRNA and protein levels of *Cav*1.2 and *Cav*1.3, the α1 subunits of L-type VGCCs, in *Pja1*-deficient CA1 (Figure 4D–F; *Cav1*.2 mRNA: **** *p* < 0.0001; *Cav*1.3 mRNA: *** *p* < 0.0005; *Cav1*.2 protein: ** *p* < 0.01; *Cav1*.3 protein: * *p* < 0.05).

### 2.5. PRAJA1 Deficiency Enhances Specific Aspects of Learning and Memory

**Figure 5:**
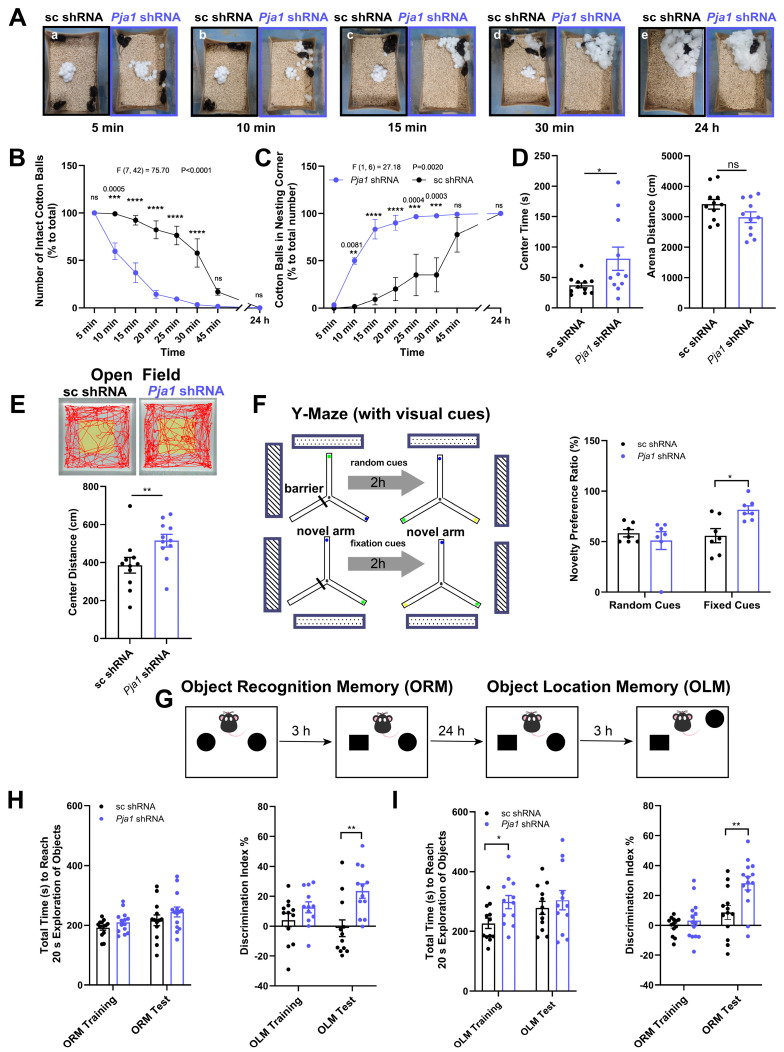
*Pja1* deficiency in the CA1 region alters nest-building behavior. (**A**) Snapshots illustrating accelerated nest-building progression in *Pja1* knockdown mice over time (a: 5 min, b: 10 min, c: 15 min, d: 30 min, e: 24). Four cages with four mice each were used per group (sc shRNA and *Pja1* shRNA). Throughout the figures, black squares and circles indicate sc shRNA data and blue squares and circles indicate *Pja1* shRNA data. (**B**) Quantification of cotton ball shredding, demonstrating increased shredding in *Pja1* knockdown mice: *n* = 4 cages/16 mice per group(ns: *p* > 0.05, *** *p* < 0.0005, **** *p* < 0.0001 (Sidak’s multiple comparisons test). (**C**) Time course of cotton ball transfer, showing significantly faster transfer in the *Pja1* shRNA group (*n* = 4 cages/16 mice per group. ns: *p* > 0.05, ** *p* = 0.0081, *** *p* < 0.0005, **** *p* < 0.0001: Sidak’s multiple comparisons test). (**D**) Open field test results, showing no difference in total ambulatory distance but reduced distance in the center zone for *Pja1* knockdown mice (Mann-Whitney test: ns: *p* > 0.05, * *p* < 0.05, *n* = 11 (sc shRNA) and *n* = 11 (*Pja1* shRNA)). (**E**) Representative movement traces and quantification of center residence time, showing a reduction in *Pja1* knockdown mice (** *p* < 0.01). (**F**) Spatial novelty Y-maze test: *Left-top:* Schematic diagram of the spatial novelty preference Y-maze test with fixed visual cues. *Left-bottom:* Schematic diagram of the Y-maze with random cues. *Right:* Novelty preference ratio during the test phase. *n* = 7 per group. * *p* < 0.05, Mann-Whitney test. The use of different cues is indicated by the colored circles in the Y-Maze arms. (**G**) Schematic diagram of the object recognition and object location memory task design. (**H**) Discrimination indices for ORM and OLM, demonstrating enhanced memory in *Pja1* knockdown mice (*n* = 13 for sc shRNA, *n* = 14 for *Pja1* shRNA. ** *p* < 0.01, Mann-Whitney test. (**I**) Exploration time during training and testing phases of ORM and OLM, showing increased time to criterion during testing in *Pja1* knockdown mice (*n* = 12 per group. * *p* < 0.05, ** *p* < 0.01, Mann-Whitney test).

Behavioral analysis revealed that *Pja1* deficiency in the CA1 region significantly accelerated nest-building activity, with *Pja1* knockdown mice utilizing a greater proportion of cotton balls and exhibiting increased shredding within the first 30 min of the test (Figure 5A–C; ** *p* < 0.01). In the open field test, while total ambulatory distance was unchanged, *Pja1* knockdown mice showed reduced ambulatory distance and residence time in the center zone (Figure 5D,E; * *p* < 0.05), suggesting altered anxiety-like behavior. Spatial memory assessment using the Y-maze with random cues showed no difference, but in the Y-maze with fixed visual cues, *Pja1* knockdown mice exhibited a significantly increased novel preference ratio, indicating improved spatial recognition memory (Figure 5F; * *p* < 0.05). Furthermore, *Pja1* knockdown significantly enhanced both object recognition memory (ORM) and object location memory (OLM), as evidenced by increased discrimination indices (Figure 5G–I; ORM: ** *p* < 0.01; OLM: ** *p* < 0.01). Interestingly, while overall exploration time was similar, *Pja1* knockdown mice took longer to reach the exploration criterion during the testing phases of both ORM and OLM (Figure 5I; * *p* < 0.05), potentially reflecting enhanced engagement with the tasks. No deficits were observed in trace fear conditioning (Figure S2B,C).

### 2.6. Consequences of Pja1 Overexpression in the Hippocampal CA1 Region

**Figure 6:**
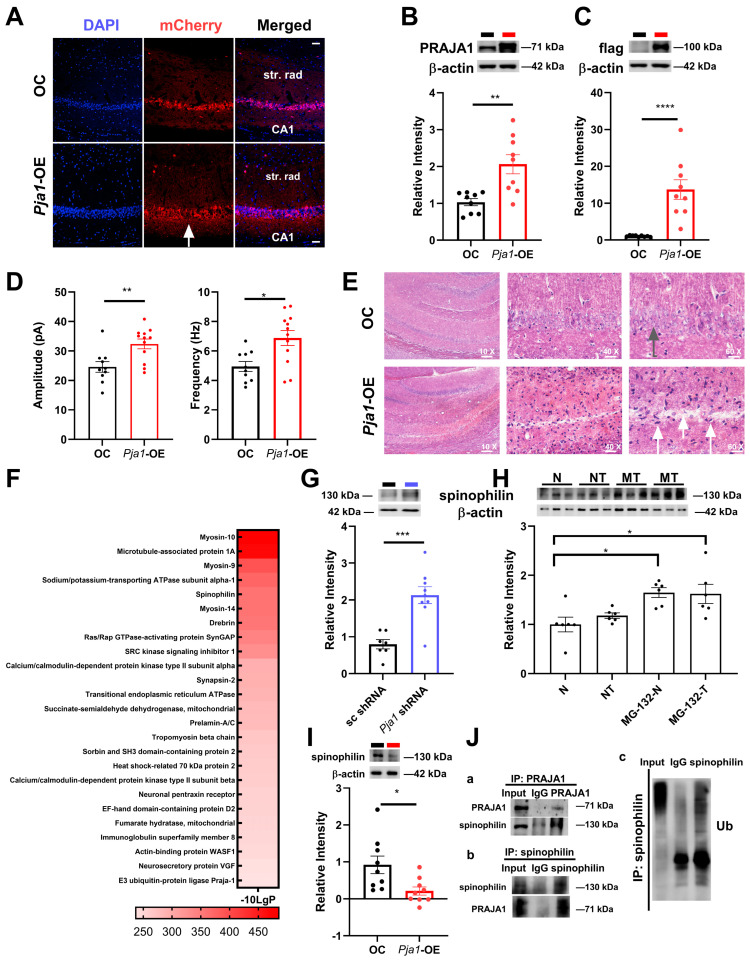
PRAJA1 overexpression in the hippocampal CA1 region induces neuronal and synaptic alterations. (**A**) *Pja1* Overexpression: Representative immunofluorescence images of the CA1 region showing mCherry (red, indicating *Pja1* expression) and DAPI (blue, nuclear stain) in mice injected with control virus (OC, top row) or *Pja1*-OE virus (bottom row). Scale bar: 50 μm. (**B**,**C**) Confirmation of overexpression: Western blot analysis of *Pja1* and FLAG tag (confirming the expression of the *Pja1*-OE construct) levels in the hippocampal CA1 region. OC: *n* = 9; *Pja1*-OE: *n* = 9; ** *p* < 0.01, **** *p* < 0.0001. (**D**) mIPSC frequency and amplitude: Quantification of mIPSC frequency and amplitude in CA1 pyramidal neurons (*Pja1*-OE results are displayed or labeled in red color for symbols, bars and lines and in black color for OC or sc shRNA groups). Amplitude, *Pja1*-OE: 32.39 ± 1.64, *n* = 12 cells, OC: 24.58 ± 1.79, *n* = 10 cells, * *p* < 0.05; frequency: ** *p* < 0.01; N = 3 mice per group, unpaired *t*-test). (**E**) Calcium Staining: Von Kossa staining of the CA1 region revealed increased intracellular calcium deposition in cells (white arrows on dark blue cells) and cell death (one arrow on white region) in *Pja1*-OE mice. The stratum pyramidale layer remains intact in OC mice (green arrow). (**F**) Ranking of 25 identified proteins specifically associated with PRAJA1 in the hippocampus on the basis of the −10-log *p*-value according to the mass spectrometry data. These identified proteins were screened on the basis of intensity and enrichment ratio. (**G**–**I**) Spinophilin protein levels: Western blot analysis showing spinophilin protein levels in the CA1 region under control conditions (OC & sc shRNA), (**G**) *Pja1* shRNA vs. sc shRNA, *Pja1* shRNA 2.13 ± 0.23, *n* = 9; sc shRNA 0.80 ± 0.16, *n* = 7; *** *p* < 0.001 (WB lanes: sc shRNA: black line; *Pja1* shRNA: blue line). (**H**) *Pja1*-OE + MG-132, and OC + MG-132 conditions (* *p* < 0.05, Sidak’s multiple comparisons test, *n* = 6 per group, N: no drug, normal conditions; NT: no drug, but STET; MG-132-N (MN): MG-132; MG-132-T (MT): MG-132 and STET). (**I**) *Pja1*-OE vs. OC (*n* = 9 per group). * *p* < 0.05. (**J**) Co-immunoprecipitation analyses: (**a**) Co-immunoprecipitation experiments confirming the interaction between PRAJA1 and spinophilin, and demonstrating ubiquitination of spinophilin. Bands of spinophilin at the position corresponding to 130 kDa were detected in the immunoprecipitates obtained with the antibody against PRAJA1 in CA1 extracts. (**b**) Bands of PRAJA1 at the position corresponding to 130 kDa were detected in the immunoprecipitates obtained with the antibody against spinophilin in CA1 extracts in Co-IP experiments. (**c**) Bands of ubiquitin (K48) at the position corresponding to 130 kDa were detected in the immunoprecipitates obtained with the antibody against spinophilin in CA1 extracts in Co-IP experiments.

To investigate the opposing effects of altered PRAJA1 levels, we overexpressed PRAJA1 in the CA1 region using an AAV9 vector (*Pja1*-OE; Figure 6A–C). Western blot analysis confirmed a significant increase in both PRAJA1 and FLAG-tagged *Pja1*-OE protein levels compared to control virus-injected mice (Figure 6B; PRAJA1: *Pja1*-OE: 2.06 ± 0.26; OC: 1.03 ± 0.09, ** *p* < 0.01; FLAG: *Pja1*-OE: 13.73 ± 2.67; OC: 1.02 ± 0.07, *** *p* < 0.0005, *n* = 9 per group). Strikingly, over half of the *Pja1*-overexpressing mice exhibited seizure-like behaviors (Figure S3), indicating a potential disruption of neuronal excitability. Consistent with this, electrophysiological recordings revealed a significant increase in the frequency of mIPSCs in *Pja1*-OE neurons compared to controls (Figure 6D; frequency: *Pja1*-OE: 6.88 ± 0.51 Hz, *n* = 12 cells; OC: 4.95 ± 0.33 Hz, *n* = 10 cells, ** *p* < 0.01), demonstrating a bidirectional modulation of inhibitory synaptic transmission by PRAJA1 levels. Furthermore, Von Kossa staining revealed a substantial increase in calcium deposition within the CA1 pyramidal cell layer of *Pja1*-OE mice, with a notable absence of staining in the nucleus (Figure 6E), suggesting potential neuronal damage or dysfunction due to calcium overload.

Given PRAJA1’s function as an E3 ubiquitin ligase, and our identification of spinophilin as a PRAJA1 interactor, we investigated if spinophilin is a PRAJA1 substrate. Consistent with this, spinophilin protein levels were significantly increased in *Pja1*-knockdown mice (Figure 6G; *Pja1* shRNA: 2.13 ± 0.23; sc shRNA: 0.80 ± 0.16) and significantly decreased following PRAJA1 overexpression (Figure 6I; *Pja1*-OE: 0.21 ± 0.11, OC: 0.92 ± 0.24, ** *p* < 0.01). Importantly, treating hippocampal slices with the proteasome inhibitor MG132 increased spinophilin levels under both basal and LTP-inducing conditions (Figure 6H), and co-immunoprecipitation confirmed the interaction between PRAJA1 and spinophilin (Figure 6J), together indicating that spinophilin is a substrate of PRAJA1.

### 2.7. Behavioral Consequences of PRAJA1 Overexpression in the Hippocampal CA1 Region

**Figure 7:**
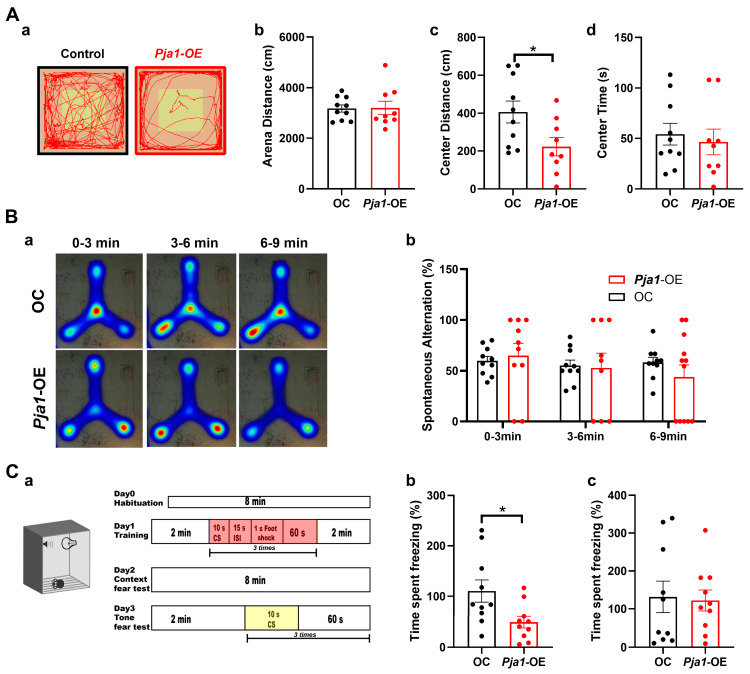
Behavioral Characterization of Mice with PRAJA1 Overexpression in CA1. (**A**) Open field test results: (**a**) Representative movement traces (**b**) No significant difference in total ambulatory distance (*Pja1*-OE: 3202 ± 265.5 cm, *n* = 9; OC: 3181 ± 143.7 cm, *n* = 10; *p* > 0.05, Mann-Whitney test); (**c**) Significantly reduced ambulatory distance in the center zone for *Pja1*-OE mice (* *p* < 0.05, Mann-Whitney test); (**d**) No significant difference in center residence time. (*Pja1*-OE: 46.46 ± 12.68 cm, *n* = 9; OC: 54.21 ± 10.78 cm, *n* = 10; *p* > 0.05, Mann-Whitney test). (**B**) Y maze test: (**a**) Heatmaps of mouse movement in a video-imaged Y-maze with three arms, each 35 cm long. (**b**) spontaneous alternation ratio in the Y-maze, 0–3 min: *Pja1*-OE: 64.98 ± 12.07, OC: 59.76 ± 4.44, *n* = 10 for each group; *p* > 0.05; 3–6 min: *Pja1*-OE: 50.83 ± 13.16, OC: 55.18 ± 5.37, *p* > 0.05; 6–9 min: *Pja1*-OE: 40.03 ± 14.10, OC: 58.22 ± 5.05, *n* = 10 per group; *p* > 0.05 Mann-Whitney test. (**C**) Trace fear conditioning: (**a**) schematic and workflow of the trace fear conditioning test, (**b**) percentage of freezing time during the context fear test. *Pja1*-OE: 48.97 ± 11.42, OC: 110.4 ± 21.99, *n* = 10; * *p* < 0.05, Mann-Whitney test. (**c**) Percentage of freezing time during the tone fear test, *Pja1*-OE: 122.3 ± 27.64, OC: 131.6 ± 41.58, *n* = 10, Mann-Whitney test.

In contrast to the effects of *Pja1* knockdown, overexpression of PRAJA1 in the CA1 region resulted in distinct behavioral alterations. Open field testing revealed that *Pja1*-OE mice exhibited a significantly shorter ambulatory distance in the center zone compared to controls (Figure 7(Ac); *Pja1*-OE: 222.7 ± 48.4 cm, *n* = 9; OC: 406 ± 58.5 cm, *n* = 10; * *p* < 0.05, Mann-Whitney test), suggesting increased anxiety-like behavior. Furthermore, *Pja1*-OE mice showed increased variability in Y-maze exploration, with a tendency to remain in the corners more often than control mice (Figure 7B; *p* >0.05). Notably, a substantial proportion of *Pja1*-OE mice failed to meet the exploration criteria in both the object recognition and object location memory tasks (Tables S1 and S2). Additionally, *Pja1*-OE mice displayed impaired trace fear memory, as demonstrated by significantly reduced freezing behavior during the context test (Figure 7(Cb); *Pja1*-OE: 48.97 ± 11.42%, *n* = 10; OC: 110.4 ± 22%, *n* = 10, * *p* < 0.05), indicating impaired hippocampus-dependent associative learning.

### 2.8. Exploring the Role of PRAJA1 in an Alzheimer’s Disease-like Animal Model

**Figure 8:**
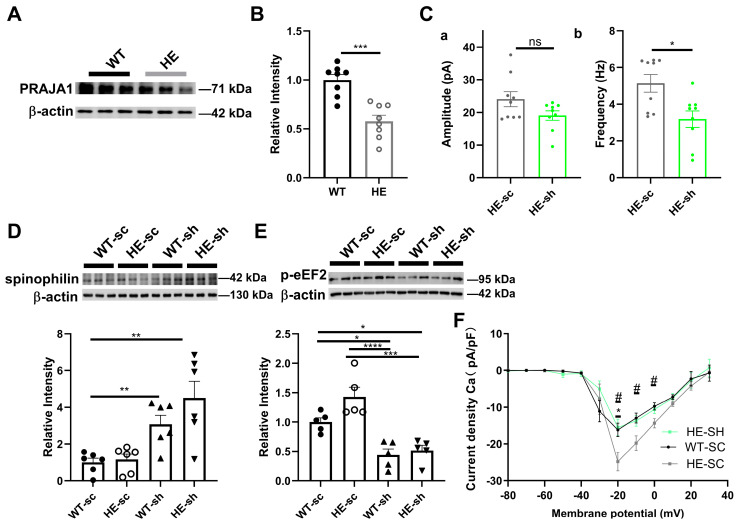
PRAJA1 and its Interacting Partners Spinophilin in the 5xFAD Mouse Model of Alzheimer’s Disease. (**A**) Representative Western blots showing reduced PRAJA1 protein levels in the CA1 of 5xFAD mice. (**B**) Quantification of PRAJA1 protein levels, demonstrating a significant reduction in 5xFAD mice. *** *p* = 0.0002, unpaired *t* test. (**C**) Quantification of (**b**) mIPSC frequency in 5xFAD mice with *Pja1* knockdown, showing a significant decrease in the knockdown group, while significant changes for (**a**) amplitude were not detectable (*n* = 9 cells per group, * *p* > 0.05, Mann-Whitney test). (**D**) Western blot analysis and quantification of spinophilin levels in 5xFAD mice with *Pja1* knockdown, showing significantly increased levels in the knockdown group (*n* = 6 per group; ** *p* < 0.01, Sidak’s test). (**E**) Western blot analysis and quantification of p-eEF2 levels in 5xFAD mice with *Pja1* knockdown, showing significantly decreased levels in the knockdown group (*n* = 5 per group, * *p* < 0.05, *** *p* < 0.0005, **** *p* < 0.0001, Sidak’s test). (**F**) I–V curves of VGCC currents in 5xFAD mice with *Pja1* knockdown, showing suppressed currents in the knockdown group (*n* = 12 cells per group; * *p* < 0.05, ^#^ *p* < 0.05, Sidak’s test.

Given the role of PRAJA1 in synaptic function and plasticity, and reports of altered PRAJA1 expression in Alzheimer’s disease (AD) patients [20], we examined its involvement in AD-related synaptic dysfunction using the 5xFAD mouse model. Consistent with previous findings, we observed a significant decrease in PRAJA1 protein levels in the hippocampus of 5xFAD mice compared to wild-type controls (Figure 8A,B; WT: 1 ± 0.55; HE: 0.58 ± 0.06; *n* = 8 per group, *** *p* = 0.0002). Further knockdown of PRAJA1 in 5xFAD mice resulted in a significant decrease in the frequency of mIPSCs in CA1 neurons (Figure 8Cb; HE-sc shRNA: 5.14 ± 0.48 Hz; HE-*Pja1* shRNA: 3.19 ± 0.45 Hz; *n* = 9 cells per group, * *p* < 0.05), suggesting that reduced PRAJA1 may contribute to the elevated mIPSC frequency typically observed in this AD model. *Pja1* knockdown in 5xFAD mice also led to a significant decrease in the levels of phosphorylated eukaryotic elongation factor 2 (p-eEF2) (Figure 8E). Interestingly, despite no significant difference in basal spinophilin levels between wild-type and 5xFAD mice, *Pja1* knockdown in the 5xFAD background significantly increased spinophilin protein levels (Figure 8D). Finally, while AD mice show an age-related increase in VGCC density, further knockdown of *Pja1* in 5xFAD mice suppressed VGCC currents (Figure 8F; * *p* < 0.05 for WT vs. HE, ^#^ *p* < 0.05 for HE sc shRNA vs. HE *Pja1* shRNA).

Publicly available data from the AlzData database (Available online: http://www.alzdata.org/; accessed on 7 January 2025) also revealed a decreasing trend in PRAJA1 expression in AD [22,23]. These results suggest a potential connection between the downregulation of PRAJA1 and AD progression. Taken together, these results suggest that reduced *Pja1* expression in AD might represent a compensatory mechanism. Further investigations into the complex interplay between *Pja1* and its interacting partners (such as spinophilin and VGCCs) and the molecular mechanisms underlying AD pathology are warranted.

## 3. Discussion

This study unveils PRAJA1 as a critical and multifaceted regulator of synaptic architecture, transmission, and cognitive function within the hippocampus, thereby revealing its significant role in shaping neuronal circuits.

### 3.1. Pja1 Regulates Synaptic Protein Expression and Density

Our findings demonstrate that while PRAJA1 protein levels remain stable in the CA1 region across the lifespan (Figure 1), its targeted manipulation exerts profound effects on synaptic structure and function. Specifically, PRAJA1 knockdown triggers a coordinated downregulation of key synaptic proteins, including PSD-95, SNAP-25, and Munc18-1 (Figure 2E), resulting in reduced spine density (Figure 2D) and compromised excitatory and inhibitory synaptic transmission. SNAP-25 and Munc18-1 are critical components of the SNARE complex, a core machinery for neurotransmitter release, including both glutamate and GABA [24,25]. Munc18-1, which acts as a template for SNARE assembly, is essential for vesicle priming and fusion [24], while SNAP-25 facilitates membrane fusion within the complex. Consistent with our findings, studies in the hippocampus show that reduced expression of Munc18-1 or SNAP-25 impairs synaptic transmission and neurotransmitter release, particularly in the CA1 region [25]. Such perturbations of SNARE function have been implicated in several neurological disorders, including “SNAREopathies” such as epilepsy and intellectual disability [25,26]. This underscores PRAJA1’s fundamental role in maintaining synaptic integrity and the delicate excitatory/inhibitory balance within hippocampal circuits. The concomitant increase in spinophilin (neurabin 2) levels following PRAJA1 knockdown further highlights its role in regulating spine dynamics, aligning with previous reports [27] and suggesting an evolutionarily conserved mechanism of synaptic remodeling. The *Pja1*-mediated regulation of PSD-95 is therefore a likely mechanism contributing to reduced excitatory synaptic transmission, and the increased spinophilin levels upon *Pja1* depletion are consistent with its established function in restricting spine density [27].

### 3.2. Pja1 Critically Influences Synaptic Transmission and Neuronal Excitability

The functional consequences of PRAJA1 deficiency extend to significant alterations in synaptic transmission. The reduced NMDA/AMPA ratio following PRAJA1 knockdown indicates a weakening of excitatory drive, likely underpinned by the decreased expression of key excitatory receptor subunits *Gria1/2* and *Grin2a/b* (Figure S1A) [28]. Furthermore, the PRAJA1-mediated ubiquitination of spinophilin suggests that its increased levels upon *Pja1* knockdown could influence AMPA and NMDA receptor activity through PP1 interaction [27], potentially exacerbating the observed reduction in excitatory transmission. Conversely, the decrease in mIPSC amplitude and frequency (Figure 3B,C) reveals compromised GABAergic inhibition, potentially resulting from a loss of GABAergic synapses or interneurons, or reduced expression of inhibitory synaptic genes like *Gabra1* and *Gabrb2* (Figure S1B). The downregulation of presynaptic proteins may impair vesicle release machinery [28], directly impacting mIPSC frequency Notably, our discovery that PRAJA1 regulates L-type voltage-gated calcium channels (L-VGCCs) reveals a crucial role for this E3 ligase in modulating neuronal excitability. The significant decrease in L-VGCC currents and *Cav1.2*/*Cav1.3* mRNA levels (Figure 4D,F) following PRAJA1 knockdown suggests its involvement in controlling calcium influx, a critical signaling pathway for dendritic development, neuronal survival, and synaptic plasticity [29]. Given the well-established link between calcium dysregulation and neurodegenerative processes, particularly in Alzheimer’s disease [30,31,32,33], this positions PRAJA1 as a key determinant of neuronal vulnerability, and its modulation of L-VGCCs represents a potential therapeutic avenue. While the precise mechanism of *Pja1*-mediated L-VGCC regulation requires further elucidation, the implication of increased VGCC activity and calcium influx in age-related neuronal dysfunction and neurotoxic vulnerability [34] further highlights the therapeutic relevance of this pathway.

### 3.3. Pja1 Acts as a Constraint on Specific Hippocampus-Dependent Memory Processes

Strikingly, despite the observed reductions in synaptic transmission, PRAJA1 deficiency paradoxically enhanced performance in several hippocampus-dependent memory tasks, including object recognition memory (ORM), object location memory (OLM), and the Y-maze with fixed visual cues (Figure 5F–I) [35,36,37]. This enhanced memory performance, alongside the observed accelerated nest-building behavior (Figure 5A), suggests that PRAJA1 normally acts as a constraint on specific memory processes and innate behaviors associated with the CA1 region. The downregulation of PRAJA1 may therefore create a more permissive environment for synaptic plasticity and memory encoding [38]. The observed reduction in phosphorylated eEF2 (p-eEF2) levels (Figure 8E), a known inhibitor of memory consolidation [39,40], following PRAJA1 knockdown provides a potential molecular mechanism for this enhancement. The specificity of this memory enhancement is highlighted by the finding that no changes were detected in the Y-maze test with random cues (Figure 5F), suggesting the improved performance is specific to tasks relying on hippocampal processing. The opposing behavioral effects observed with PRAJA1 overexpression further validate its critical role in maintaining the delicate balance required for optimal cognitive function. This finding also offers a compelling interpretation for the observed downregulation of PRAJA1 in Alzheimer’s disease models (Figure 8A), suggesting it may represent an endogenous compensatory mechanism aimed at counteracting synaptic dysfunction and promoting plasticity.

### 3.4. Pja1 and Spinophilin: A Critical Link to Alzheimer’s Disease Pathogenesis

These findings have significant implications for understanding the molecular mechanisms underlying both normal memory function and the pathogenesis of Alzheimer’s disease. Consistent with the complex expression landscape often observed in advanced AD models, our results demonstrate a significant decrease in PRAJA1 expression within the hippocampus of 5xFAD mice, further highlighting the relevance of PRAJA1 dysregulation in AD pathology. The involvement of spinophilin, a key regulator of dendritic spine structure and plasticity highly enriched in the hippocampus [27,41,42], is particularly noteworthy. Our data suggest that the downregulation of PRAJA1 in AD may not simply be a consequence of neurodegeneration but could represent an active attempt by the neuronal network to enhance plasticity and compensate for synaptic deficits. This is further underscored by the consistent downregulation of spinophilin in both 5xFAD mice and the brains of individuals with cognitive impairment [43,44], with its levels in the hippocampal CA1 region showing a significant negative correlation with Braak NFT staging and clinical severity [44]. Interestingly, while global spinophilin levels were not significantly altered in our 5×FAD cohort—a phenomenon that may be due to age-related UPS dysregulation masking direct in vivo relationships—*Pja1* knockdown consistently induced a pronounced increase in spinophilin expression in both wild-type and 5×FAD backgrounds. This observation highlights the critical role of PRAJA1 as a regulator of spinophilin expression. The identification of spinophilin as a direct PRAJA1 target provides a compelling mechanistic link between PRAJA1, synaptic remodeling, and AD pathology. As an E3 ligase, PRAJA1 likely regulates spinophilin stability through ubiquitination, potentially via recognition of its PDZ domain [45]. The observed downregulation of spinophilin in AD, therefore, might be a consequence of altered PRAJA1 activity. Thus, our study positions PRAJA1, along with its downstream targets like p-eEF2, spinophilin, and L-VGCCs, as a compelling therapeutic target for cognitive enhancement in AD and potentially other neurological disorders [40].

### 3.5. Translational Relevance of PRAJA1

In interpreting the results of this study, it is important to consider the existing phenotypic landscape of *Pja1* disruption. While constitutive *Pja1* knockout mice exhibit a limited phenotype, primarily reduced locomotor activity in females (MGI:1101765), and current Gene Ontology annotations lack synaptic plasticity terms for *PRAJA1* (Q8NG27), our acute manipulation studies reveal a robust and previously unappreciated role for *PRAJA1* in regulating synaptic plasticity and hippocampus-dependent memory. This apparent divergence is likely multifactorial, with developmental adaptation in germline knockout models potentially masking the acute synaptic functions we observe in adult animals. Furthermore, although current human genetic databases, such as GeneCards and NCBI, document single nucleotide variants in *PRAJA1*, robust associations with major neurodegenerative or memory-related phenotypes remain limited. However, GeneCards’ GWAS catalog highlights an intriguing association between *PRAJA1* and schizophrenia [46], a complex neuropsychiatric disorder with established synaptic underpinnings. This emerging human genetic association, when considered in conjunction with our novel mouse findings, underscores the translational relevance of *PRAJA1* in neuronal function and dysfunction. The absence of a specific Mendelian Inheritance in Man (MIM) phenotype code for *PRAJA1* underscores the notion that its precise role in human disease is still emerging and warrants further investigation. We hypothesize that such adaptive processes, possibly involving functional redundancy with other *PRAJA* family members, may also contribute to the subtle human phenotypic landscape observed to date.

### 3.6. Unresolved Questions and Future Directions

While our study provides compelling evidence for the role of PRAJA1 in regulating synaptic plasticity and memory, several important questions remain. Elucidating the precise molecular mechanisms by which PRAJA1 regulates the expression and function of its various synaptic targets, including PSD-95 and L-VGCCs, warrants further investigation. Future studies should explore the specific signaling pathways involved and identify additional PRAJA1 substrates in the hippocampus. Furthermore, investigating the dynamic regulation of PRAJA1 expression and activity in response to different physiological and pathological stimuli will be crucial for fully understanding its role in neuronal function. Finally, exploring the therapeutic potential of modulating PRAJA1 activity in preclinical models of AD and other cognitive disorders holds significant promise. Investigating the dynamic regulation of these processes in different physiological and pathological contexts could uncover novel therapeutic avenues for treating neurological disorders.

## 4. Materials and Methods

### 4.1. Animals

C57BL/6 mice (male, 8–10 weeks old, 20–22 g) were obtained from Jie Si Jie Experimental Animal Co., Ltd. (Shanghai, China), and adult male heterozygous (HE) 5xFAD mice and their wild-type (WT) littermates were obtained from Shanghai Model Organisms Center, Inc. (Shanghai, China). All the mice were maintained following the established standards of animal care and procedures of the Institutes of Brain Science and State Key Laboratory of Medical Neurobiology (Approval No. 20210302-129, and date of approval: 2 March 2021; Fudan University, Shanghai, China). In addition, all experimental protocols were approved by the Medical Experimental Animal Administrative Committee of Fudan University (Shanghai, China). Animals were housed with consistent group sizes and environmental conditions (temperature, humidity, light cycle) throughout the study. All groups received identical access to standard rodent chow and water and were subjected to the same maintenance protocols. Initial group allocation was blinded to the experimenter, who was then informed of the group allocation prior to the start of treatment. Efforts were made to minimize the number of animals used.

### 4.2. Stereotaxic Intrahippocampal Injections

Stereotactic intrahippocampal microinjections were performed as described previously [47,48,49]. Briefly, the mice were anesthetized via the intraperitoneal administration of 2.5% tribromoethanol (Avertin, injected: 0.1 mL/10 g) and 3 mg/mL xylazine (injected: 0.04 mL/10 g) and then secured in a stereotaxic frame [49] to allow precise insertion of glass pipettes into the intermediate hippocampus. The CA1 area (AP −3.60 mm, ML 3.4 mm, DV −3.65 mm) was infected with viruses through a glass micropipette connected to a paraffin oil-filled microsyringe and tube. Depth of anesthesia during stereotaxic injections was monitored by assessing respiratory rate and reflexes (eyelid, paw/tail pinch). Stable respiration and absence of reflexes indicated adequate anesthesia; additional anesthetic was administered if reflexes were present.

Virus information. The adeno-associated virus AAV9-hSyn-*Pja1*-mCherry-3flag and its control virus, and the lentivirus were packaged by Shanghai Shengbo (Shanghai, China). The plasmid containing the *Pja1* shRNA in lentivirus was a gift from Prof. Oliver Stork (Otto-von-Guericke University Magdeburg, Germany). The short hairpin RNA targeting the sequence of mouse *Pja1* (NCBI accession no. XM_011247544 [50]) was designed as follows: 5′ GCACAGATCAACTGCCAATCA-3′. The sequence of the scrambled control shRNA was 5′TTCTCCGAACGTGTCACGT-3′. The lentiviral vectors used were based on the vector backbone pLL3.7.

### 4.3. Reverse Transcription Quantitative Polymerase Chain Reaction

The CA1 area was isolated from snap-frozen acute transverse hippocampal slices (350 µm) in ice-cold PBS. Total RNA was extracted via the TaKaRa MiniBEST Universal RNA Extraction Kit (#9767, Otsu, Shiga,, Japan) according to the manufacturer’s instructions. Quantitative real-time polymerase chain reaction (Q-PCR) was performed using the PrimeScript™ RT Reagent Kit with gDNA Eraser (#RR047A, Otsu, Shiga,, Japan), and mRNA levels were measured using TB Green^®^ Premix Ex Taq™ II (#RR820A, Otsu, Shiga, Japan). A QuantStudio 3 Real-Time PCR system was used for the RT-qPCR assays (Thermo Fisher Scientific, Waltham, MA, USA). Relative mRNA levels were normalized to Ct values according to the 2-ΔΔct calculation method. The sequences of the primers (Sangon Biotech, Shanghai, China) used in this study are as follows in the following Table 1.

### 4.4. Western Blot Analysis

Western blot analysis was conducted following previously described procedures [51,52]. The CA1 tissue samples were collected in a similar manner as those used for the q-PCR analyses described above. Whole-cell lysates were extracted from CA1 tissue via RIPA buffer with a protease and phosphatase inhibitor cocktail (Beyotime Institute of Biotechnology, Haimen, Jiangsu Province, China). The protein concentrations were determined using a bicinchoninic acid (BCA) assay kit (Beyotime Institute of Biotechnology, Haimen, Jiangsu Province, China), after which the proteins were denatured at 95 °C. The protein samples were denatured and separated via 8% sodium dodecyl sulfate-polyacrylamide gel electrophoresis (SDS–PAGE). The proteins were subsequently transferred to a polyvinylidene fluoride (PVDF) membrane (Immobilon-P, Millipore, Billerica, MA, USA), which was subsequently blocked with 5% nonfat milk at room temperature. Primary antibodies against PRAJA1 were incubated with the samples overnight at 4 °C. The membranes were incubated with horseradish peroxidase-conjugated anti-rabbit secondary antibodies for one hour at room temperature. The bands were then visualized using the eBlot Touch Imager (eBlot, Shanghai, China). The Western blot results were quantified via AlphaView software (v: 3.5.0). The quantified values of each blot were normalized to the loading control amounts. The Western blot data are expressed as the means ± standard errors of the means (SEMs). Statistical analysis was performed as described in the text.

The following antibodies were used: PRAJA1 (1:1000, ProteinTech, Chicago, IL, USA, 17687-1-AP), actin (1:1000, Cell Signaling, Danvers, MA, USA, CST:4970s), Cav1.2 (1:500, Alomone, Jerusalem, Israel, ACC-003), Cav1.3 (1:500, Alomone, Jerusalem, Israel, ACC-005), SNAP25 (1:1000, CST:5309s), Munc18-1 (1:1000, CST:d406v), rabbit anti-PSD-95 (1:1000, CST:2507s), spinophilin (1:1000, CST:14136s), and synapsin (1:1000, CST:2312s). p-synapsin (1:1000, Cell Signaling, Danvers, MA, USA, CST:2311s), syntaxin 1A (1:1000, CST: 13002s).

### 4.5. Immunofluorescence and Von Kossa’s Staining

The mice were deeply anesthetized, perfused with 4% paraformaldehyde, and fixed in 4% paraformaldehyde in PBS overnight. The brains were then transferred to a 30% sucrose solution and sectioned at 30 µm using a cryostat (Leica 1900, Leica, Wetzlar, Germany). The sections were mounted on glass slides for immunofluorescence. The sections were permeabilized with 0.3% Triton X-100 for 30 min, followed by blocking with 10% goat serum for 2 h at room temperature. The sections were incubated overnight at 4 °C with an anti-GFP primary antibody (1:200; Abcam, Cambridge, UK, ab13970t). After three washes in PBS, the sections were incubated for 2 h with secondary species-specific antibodies: goat-anti-chicken (488, Abcam, ab150169) and DAPI (405, Roche, Basel, Switzerland, 10236276001).

The dendritic spines were imaged using a Nikon A1 confocal microscope and a Nikon AX NSPARC confocal microscope (Tokyo, Japan). High-resolution z-stack images of the dendritic segments were acquired with a 60× oil-immersion objective. The number and density of dendritic spines were then quantified from the confocal image stacks via ImageJ (v: 1.54f), to obtain detailed morphometric measurements of the spines.

Von Kossa’s stain was used to detect calcium. The tissue samples were cut into 10-micrometer sections and sent to POWERFUL BIOLOGY (Shanghai, China) for further staining.

### 4.6. Coimmunoprecipitationh

Hippocampal CA1 tissues were lysed in RIPA buffer containing protease inhibitors (Beyotime Institute of Biotechnology, Haimen, Jiangsu Province, China). The CA1 lysates were resolved with control agarose resin (26149, Thermo-Fisher Scientific, Waltham, MA, USA). The PRAJA1 and IgG antibodies (ProteinTech, Tokyo, Japan) were immobilized in the resin and then incubated with the precleared CA1 lysates overnight. The final samples were washed from the antibody-coupled resin and boiled in lane marker sample buffer with 5× DTT. The subsequent procedure was similar to that of Western blot analysis.

### 4.7. Coimmunoprecipitation with Subsequent Mass Spectrometry

The samples obtained from the co-immunoprecipitation method were sent to the Core Facility of Shanghai Medical College, Fudan University, for mass spectrometry analysis.

DTT was added to the samples and reacted at 37 °C for 1 h, then IAA was added, and after half an hour of reaction at room temperature and shielded from light, 4 times the volume of ice acetone was added and incubated at −80 °C overnight. The next day, the supernatant was collected after centrifugation and proteins were enzymatically digested. Samples were reconstituted with 200 μL ammonium bicarbonate (50 mM) and trypsin was added at a ratio of 1:50. The samples were digested overnight at 37 °C, followed by desalting using a Waters Sepak desalting column in preparation for mass spectrometric analysis.

NanoElute (Bruker Daltonics, Osaka, Japan) liquid chromatography was used for chromatographic separation, using a 250 mm × 75 μm column (InoOpticks), after which 200 ng of peptides were analyzed on a machine (TIMS-TOF Pro mass spectrometer, Bruker Daltonics, Osaka, Japan). Then the data were analyzed by Peaks Online (Bioinformatics Solutions Inc. Vesion 1.7, Waterloo, ON, Canada), and searched against the swissprot Mus musculus database (17.046 proteins, downloaded 16 April 2021). The peak areas of the retrieved proteins were used for subsequent statistical analysis. The experimental results were analyzed online using websites such as DAVID [14].

### 4.8. Field Potential Recordings

The acute hippocampal slices were prepared as previously reported [52,53,54,55]. Field excitatory postsynaptic potentials (fEPSPs) were evoked by stimulating Schaffer-collateral fibers of the stratum radiatum (biphasic rectangular current pulses, 100 µs/polarity) and recorded using a differential amplifier (EXT-20F; lowpass filter: 3 kHz, high-pass filter: 0.1 Hz; npi electronic GmbH, Tamm, Germany) [52,56]. The recorded field potentials were then digitized at a sampling frequency of 10 kHz by a CED 1401 plus AD/DA converter (Cambridge Electronics Design, Cambridge, UK). Short-term potentiation (STP) of fEPSPs was induced by weak tetanization containing 30 stimuli at 100 Hz; long-term potentiation (LTP) was induced by strong tetanization consisting of three 1-s trains at 100 Hz every 10 min [57]. The slope of the fEPSP was used as a measure of the strength of synaptic transmission. fEPSP slopes were normalized to the average baseline fEPSP slopes. The stimulation strengths were adjusted to 40–50% of the maximum fEPSP slope values.

### 4.9. Whole-Cell Voltage-Clamp Recordings

After the transfer of the incubated 350 µm thick hippocampal slices to a submerged recording chamber system (RC-26GLP; Warner, Hamden, CT, USA), the neurons were visualized using an infrared (IR)-differential interference contrast (DIC) microscope (Nikon, Tokyo, Japan) with a 40× water-immersion objective. Whole-cell voltage-clamp recording was used to study the properties of synaptic transmission in CA1 neurons. The pipette resistance ranged from 3–6 MΩ when the pipette mixture contained containing cesium methanesulfonate (110 mM), CsCl (10 mM), MgCl_2_·6H_2_O (2 mM), EGTA (0.5 mM), ATP-Na_2_ (4 mM), GTP-Na (0.4 mM), and HEPES (10 mM), pH 7.25, adjusted with CsOH (280–300 mOsm). The signals from the CA1 neurons were acquired using a MultiClamp 700B amplifier, then low-pass filtered with a 2.8 kHz Bessel filter, digitized via Digidata 1440A and acquired using Clampex 10.2 software (Molecular Devices, Sunnyvale, CA, USA). The amplitude and frequency were analyzed using MiniAnalysis 6 (Synaptosoft Inc., Fort Lee, NJ, USA).

To prevent action potential-driven transmitter release and excitatory transmission for miniature inhibitory post-synaptic currents (mIPSCs), tetrodotoxin (TTX, 1 µM, 1069, Tocris, Bristol, UK), CNQX (25 µM, C239, Sigma, Beijing, China) and DNQX (20 µM, HY-15067, MCE, Shanghai, China) were added to the ACSF.

A glass pipette was placed in the stratum radiatum, approximately 100 μm below the recording pipettes (refer to Figure 4A). Using a stimulation isolation unit (STG 1002, eutlingen, BW, Germany), stimulation was applied to record evoked EPSCs and IPSCs. To record the paired AMPA receptors (AMPARs) that mediate evoked excitatory postsynaptic currents (e-EPSCs), we clamped the membrane potential at −70 mV and perfused it with 10 μM bicuculline. To block AMPA receptors, we added 25 μM CNQX and recorded the e-EPSCs mediated by NMDAR receptors at +40 mV. The evoked inhibitory postsynaptic currents (e-IPSCs) were recorded in the presence of 25 μm CNQX and 50 μM D-AP5. Paired signals were sequentially recorded for e-IPSCs while they were held at −70 mV, −90 mV, 0 mV, or +40 mV. The CA3 region was removed to reduce the likelihood of synchronized neuronal activity, or bursts, that may result from recurrent excitation during bicuculline perfusion (see Figure 3A,I).

Voltage-gated sodium and calcium currents were elicited by 10 mV voltage steps ranging from −80 mV to +30 mV for a duration of 100 ms. To confirm the involvement of L-type calcium channels in the observed signal, an L-type calcium channel blocker, nimodipine (10 µM, Sigma, Beijing, China, 482200), and voltage-gated sodium channel blocker, TTX (1 µM, Tocris, Bristol, UK) were used to record the voltage-gated calcium currents.

### 4.10. Nest-Building Behavior

This study analyzed the nesting ability of four mice in each cage under constant ambient temperature by observing the manipulation of thirty cotton balls (approximately 1 cm in diameter) placed in the center of the cage floor. Nesting ability was assessed by analyzing the collection of cotton balls in a nesting corner and the number of cotton balls bitten [48,58]. The quantity of cotton balls in the nesting corner was determined through visual inspection and calculated as the ratio of the number of nesting balls to the total number of balls. Similarly, the degree of crushing was estimated by calculating the ratio of the number of crushed balls to the total number of balls.

### 4.11. Open Field Test

The open field test [59,60,61,62] was used to assess locomotor activity and anxiety-like behavior in the mice. The mice were placed in a 40 × 40 × 40 cm Plexiglas arena with an open floor and walls. The central area of the arena, measuring 20 × 20 cm, was designated for testing. Following a 30-minute habituation period in the test room, the mice were allowed to freely explore the open field for 5 min under dim white light. Mouse movement and location were recorded using a video camera and EthoVision XT 16 video tracking software (v: 16.0.1538, Noldus Information Technology, Wageningen, Netherlands. ). The total distance traveled, time spent in the center, and distance traveled in the center were calculated for 5 min. The percentage of distance traveled in the center of the total movement was used as a measure of anxiety-like behavior [62,63]. The arena was cleaned with 75% ethanol before and between trials.

### 4.12. Y-Maze Test and Spatial Novelty Y-Maze Test

The experiment involved placing the mice in the central area of a Y-maze (28 cm long, 5 cm wide walls, and 9 cm high) and allowing them to move freely for 8 min while being monitored by an automated tracking system (EthoVision XT video tracking software, v: 16.0.1538, Information Technology, Wageningen, Netherlands). To minimize the effects of stress on behavior during testing, the mice were kept in the testing room for at least 30 min before testing. Scoring was based on recording each arm entry, which was defined as all four paws entering the arm. The arena was cleaned with 75% ethanol between trials. To determine the maximum number of alternations, subtract two from the total number of arm entries. To evaluate spatial working memory performance, the alternation ratio was calculated using the formula: [number of correct alternations/(total number of arm entries − 2)] × 100% [64,65,66].

The spatial novelty preference (SNP) Y-maze behavioral paradigm was conducted according to the procedure outlined in Figure 5F. Manipulation methods similar to those of the Y-maze experiment were employed, with the inclusion of additional cues positioned outside of the maze. Two conditions were established for the mice: randomized cues and fixed cues. During the exposure phase, the mice were allowed to freely explore two arms, namely the ‘start’ arm and the ‘other’ arm. Following a 2-hour period, the mice were given unrestricted access to all three arms during the test phase [35,67]. The novelty preference ratio was calculated for each arm by dividing the number of entries into the novel arm by the sum of entries into both arms, and then multiplying the result by 100%. The formula used to calculate the ratio was as follows: [novel arm/(novel arm + other arm)] × 100%.

### 4.13. Object Recognition Test and Object Location Test

In object recognition tests (ORTs), the mice were positioned facing the wall with their heads opposite the objects and allowed to explore two objects placed at the bottom of the box. After a 3-h retention interval, one of the objects in the test stage was replaced with a novel object that differed in both shape and color. The exploration of each object was timed using stopwatches for a total of 20 s. The experiment used a video tracking system to monitor exploration. The experiment ended when both objects had been explored for 20 s or when the total experiment duration exceeded 9 min without meeting the 20-s exploration criterion [68]. The discrimination index (DI) shows the difference in exploration times between familiar and novel objects. The preference index was calculated using the formula ((Time of the familiar object − Time of the novel object)/(Time of the familiar object + Time of the novel object)) × 100) [36].

The day after the OMT test, the mice underwent an object location test (OLT) in the same white box. The two objects used in the OMT test were presented again to reinforce the location memory of the mice. After a retention interval of three h, we relocated one object (refer to Figure 5G) and measured the total exploration time for both the familiar and novel object positions. We used DI to evaluate object location memory performance [68,69].

### 4.14. Trace Fear Conditioning Task

Fear conditioning was conducted in a sound-isolated chamber with an acrylic glass arena featuring a grid floor for delivering foot shocks, a loudspeaker, and a ventilator. The background noise level was 70 dB SLP, and the light intensity was less than 10 lux. The experiment was monitored using an ANY-maze video tracking system (USA) [70,71,72].

Before the training day, the mice were acclimated to the experimental room for 8 min. On the first day of training, they were exposed to three conditional stimuli, each consisting of a 10-s 10 kHz tone at 70 dB, which co-terminated with an unconditional stimulus of a 0.5 mA foot shock for one second.

The animals were given two minutes to acclimate to the chamber before each stimulus exposure, with inter-stimulus intervals of 15 s. On the second day, the mice were returned to the experimental chamber for the context fear test, and their freezing behavior was recorded for six minutes.

On the third day, the tone fear test was conducted in the same chamber but in a neutral context. After the first two minutes, their freezing behavior was monitored four times with a 10-s, 4-kHz tone separated by 60-s intervals, totaling 400 s.

The study evaluated the freezing behavior of the animals via an animal tracking system that detected immobility periods lasting one second or longer. The freezing score was calculated as a percentage of the total time spent freezing during context exposure and CS presentation.

### 4.15. Statistical Analysis

Statistical comparisons were performed using GraphPad Prism 8.0 software (GraphPad Software, San Diego, CA, USA) and the data are presented as the means ± standard errors of the means (SEMs). Sample sizes were determined to ensure sufficient statistical power to detect biologically meaningful effects, consistent with established practices in the field and supported by the literature cited herein. Nonparametric tests like the Mann-Whitney test were chosen in case of the lack of a normal distribution. The use of One-way ANOVA, Two-way ANOVA, or mixed-effects analysis with Sidak’s multiple comparisons test, and unpaired *t*-test (two-tailed), were utilized and specified in results or legends. A statistically significant difference was indicated by *p* < 0.05.

## 5. Conclusions

In conclusion, this study establishes PRAJA1 as a novel and critical master regulator of synaptic architecture, transmission, and hippocampus-dependent memory. Our findings reveal a complex and intricate interplay between PRAJA1, synaptic protein turnover, neuronal excitability, and cognitive function. The observed downregulation of PRAJA1 in AD models, coupled with the memory-enhancing effects of PRAJA1 knockdown, suggests a potential compensatory role for PRAJA1 dysregulation in neurodegenerative disease. These insights open new avenues for understanding the molecular underpinnings of memory and offer a promising new therapeutic target for the treatment of cognitive impairment.

## Figures and Tables

**Table 1:** The sequences of the primers used in this study.

Gene	Forward	Reverse
*Cav1.2*	5′-GAGACAATGTGTGGAATATGCC-3′	3′-GTAGGTAGAGTTGACCACGTAC-5′
*Cav1.3*	5′-GCACAGATGAAGCCAAAAGTAA-3′	3′-CGTTCTCACCGTTTGAATCAAT-5′
*Gapdh*	5′-AGGTCGGTGTGAACGGATTTG-3′	3′-TGTAGACCATGTAGTTGAGGTCA-5′
*Gabra1*	5′-CACCATGAGGTTGACCGTGA-3′	3′-CTACAACCACTGAACGGGCT-5′
*Gabrb1*	5′-CATAGACATGGTCTCGGAAG-3′	3′-GTCAGCTACTCTGTTGTCAA-5′
*Gabrb2*	5′-ATTTGGTGGCTCAAACGGTC-3′	3′-GAGATTTCCTCACCAGCAGGA-5′
*Gabrg2*	5′-GGAGCCGGCATCAAATCATC-3′	3′-CTTTTGGCTTGTGAAGCCTGG-5′
*Gabrd*	5′-ATACACCATGACTGTGTTCC-3′	3′-TAGGCGGATAAGCTTGTTTT-5′
*Grin1*	5′-CATCGGACTTCAGCTAATCA-3′	3′-GTCCCCATCCTCATTGAATT-5′
*Grin2a*	5′-GGCTACAGAGACTTCATCAG-3′	3′-ATCCAGAAGAAATCGTAGCC-5′
*Grin2b*	5′-TTAACAACTCCGTACCTGTG-3′	3′-TGGAACTTCTTGTCACTCAG-5′
*Gria1*	5′-GCCTTAATCGAGTTCTGCTA-3′	3′-GAATGGATTGCATGGACTTG-5′
*Gria2*	5′-AGCCTATGAGATCTGGATGT-3′	3′-GAGAGAGATCTTGGCGAAAT-5′

## Data Availability

The original contributions presented in the study are included in the article/supplementary files; further inquiries can be directed to the corresponding author.

## Supplementary Materials

The supporting information can be downloaded at https://www.mdpi.com/article/10.3390/ijms26072909/s1.

## Abbreviations

The following abbreviations are used in this manuscript:

AD	Alzheimer’s disease
APP	amyloid precursor protein
eEPSC	evoked excitatory postsynaptic current
fEPSP	field excitatory postsynaptic potential
LTP	long-term potentiation
mIPSCs	miniature inhibitory postsynaptic currents
OLM	Object location memory
ORM	Object recognition memory
PPR	paired-pulse ratio
STET	strong high-frequency stimulation
UPS	ubiquitin-proteasome system

## References

[1] Hegde A.N. (2017). Proteolysis, synaptic plasticity and memory. Neurobiol. Learn. Mem..

[2] Vinci M., Treccarichi S., Galati Rando R., Musumeci A., Todaro V., Federico C., Saccone S., Elia M., Cali F. (2024). A de novo ARIH2 gene mutation was detected in a patient with autism spectrum disorders and intellectual disability. Sci. Rep..

[3] Murata T., Suzuki E., Ito S., Sawatsubashi S., Zhao Y., Yamagata K., Tanabe M., Fujiyama S., Kimura S., Ueda T. (2008). RNA-binding protein hoip accelerates polyQ-induced neurodegeneration in Drosophila. Biosci. Biotechnol. Biochem..

[4] Liu L., Liu T.T., Xie G.G., Zhu X.Q., Wang Y. (2022). Ubiquitin ligase TRIM32 promotes dendrite arborization by mediating degradation of the epigenetic factor CDYL. FASEB J..

[5] Pavlou M.A.S., Colombo N., Fuertes-Alvarez S., Nicklas S., Cano L.G., Marin M.C., Goncalves J., Schwamborn J.C. (2017). Expression of the Parkinson’s Disease-Associated Gene Alpha-Synuclein is Regulated by the Neuronal Cell Fate Determinant TRIM32. Mol. Neurobiol..

[6] Mishra L., Tully R.E., Monga S.P., Yu P., Cai T., Makalowski W., Mezey E., Pavan W.J., Mishra B. (1997). Praja1, a novel gene encoding a RING-H2 motif in mouse development. Oncogene.

[7] Nakamura N. (2018). Ubiquitin System. Int. J. Mol. Sci..

[8] Stork O., Welzl H. (1999). Memory formation and the regulation of gene expression. Cell. Mol. Life Sci..

[9] Shin J., Mishra V., Glasgow E., Zaidi S., Chen J., Ohshiro K., Chitti B., Kapadia A.A., Rana N., Mishra L. (2017). PRAJA is overexpressed in glioblastoma and contributes to neural precursor development. Genes Cancer.

[10] Consalvi S., Brancaccio A., Dall’Agnese A., Puri P.L., Palacios D. (2017). Praja1 E3 ubiquitin ligase promotes skeletal myogenesis through degradation of EZH2 upon p38alpha activation. Nat. Commun..

[11] Sasaki A., Masuda Y., Iwai K., Ikeda K., Watanabe K. (2002). A RING finger protein Praja1 regulates Dlx5-dependent transcription through its ubiquitin ligase activity for the Dlx/Msx-interacting MAGE/Necdin family protein, Dlxin-1. J. Biol. Chem..

[12] Loch C.M., Eddins M.J., Strickler J.E. (2011). Protein microarrays for the identification of praja1 e3 ubiquitin ligase substrates. Cell Biochem. Biophys..

[13] Teuber J., Mueller B., Fukabori R., Lang D., Albrecht A., Stork O. (2013). The ubiquitin ligase Praja1 reduces NRAGE expression and inhibits neuronal differentiation of PC12 cells. PLoS ONE.

[14] Wang X., Jiang Y., Feng B., Ma X., Zhang K., Yang F., Liu Z., Yang L., Yue J., Lu L. (2023). PJA1 mediates the effects of astrocytic GPR30 on learning and memory in female mice. J. Clin. Investig..

[15] Ghosh B., Karmakar S., Prasad M., Mandal A.K. (2021). Praja1 ubiquitin ligase facilitates degradation of polyglutamine proteins and suppresses polyglutamine-mediated toxicity. Mol. Biol. Cell.

[16] Watabe K., Kato Y., Sakuma M., Murata M., Niida-Kawaguchi M., Takemura T., Hanagata N., Tada M., Kakita A., Shibata N. (2020). Praja1 RING-finger E3 ubiquitin ligase suppresses neuronal cytoplasmic TDP-43 aggregate formation. Neuropathology.

[17] Wieland I., Weidner C., Ciccone R., Lapi E., McDonald-McGinn D., Kress W., Jakubiczka S., Collmann H., Zuffardi O., Zackai E. (2007). Contiguous gene deletions involving EFNB1, OPHN1, PJA1 and EDA in patients with craniofrontonasal syndrome. Clin. Genet..

[18] Suzuki T., Suzuki T., Raveau M., Miyake N., Sudo G., Tsurusaki Y., Watanabe T., Sugaya Y., Tatsukawa T., Mazaki E. (2020). A recurrent PJA1 variant in trigonocephaly and neurodevelopmental disorders. Ann. Clin. Transl. Neurol..

[19] Stork O., Stork S., Pape H.C., Obata K. (2001). Identification of genes expressed in the amygdala during the formation of fear memory. Learn. Mem..

[20] Stein T.D., Anders N.J., DeCarli C., Chan S.L., Mattson M.P., Johnson J.A. (2004). Neutralization of transthyretin reverses the neuroprotective effects of secreted amyloid precursor protein (APP) in APPSW mice resulting in tau phosphorylation and loss of hippocampal neurons: Support for the amyloid hypothesis. J. Neurosci..

[21] Koopmans F., van Nierop P., Andres-Alonso M., Byrnes A., Cijsouw T., Coba M.P., Cornelisse L.N., Farrell R.J., Goldschmidt H.L., Howrigan D.P. (2019). SynGO: An Evidence-Based, Expert-Curated Knowledge Base for the Synapse. Neuron.

[22] Xu M., Zhang D.F., Luo R., Wu Y., Zhou H., Kong L.L., Bi R., Yao Y.G. (2018). A systematic integrated analysis of brain expression profiles reveals YAP1 and other prioritized hub genes as important upstream regulators in Alzheimer’s disease. Alzheimers Dement..

[23] Zhang D.F., Fan Y., Xu M., Wang G., Wang D., Li J., Kong L.L., Zhou H., Luo R., Bi R. (2019). Complement C7 is a novel risk gene for Alzheimer’s disease in Han Chinese. Natl. Sci. Rev..

[24] Stepien K.P., Prinslow E.A., Rizo J. (2019). Munc18-1 is crucial to overcome the inhibition of synaptic vesicle fusion by alphaSNAP. Nat. Commun..

[25] Kadkova A., Murach J., Ostergaard M., Malsam A., Malsam J., Lolicato F., Nickel W., Sollner T.H., Sorensen J.B. (2024). SNAP25 disease mutations change the energy landscape for synaptic exocytosis due to aberrant SNARE interactions. Elife.

[26] Verhage M., Sorensen J.B. (2020). SNAREopathies: Diversity in Mechanisms and Symptoms. Neuron.

[27] Feng J., Yan Z., Ferreira A., Tomizawa K., Liauw J.A., Zhuo M., Allen P.B., Ouimet C.C., Greengard P. (2000). Spinophilin regulates the formation and function of dendritic spines. Proc. Natl. Acad. Sci. USA..

[28] Lu Y.M., Mansuy I.M., Kandel E.R., Roder J. (2000). Calcineurin-mediated LTD of GABAergic inhibition underlies the increased excitability of CA1 neurons associated with LTP. Neuron.

[29] Sarkar S.N., Huang R.Q., Logan S.M., Yi K.D., Dillon G.H., Simpkins J.W. (2008). Estrogens directly potentiate neuronal L-type Ca2+ channels. Proc. Natl. Acad. Sci. USA..

[30] Anekonda T.S., Quinn J.F., Harris C., Frahler K., Wadsworth T.L., Woltjer R.L. (2011). L-type voltage-gated calcium channel blockade with isradipine as a therapeutic strategy for Alzheimer’s disease. Neurobiol. Dis..

[31] Simakova O., Arispe N.J. (2006). Early and late cytotoxic effects of external application of the Alzheimer’s Abeta result from the initial formation and function of Abeta ion channels. Biochemistry.

[32] Kuchibhotla K.V., Goldman S.T., Lattarulo C.R., Wu H.Y., Hyman B.T., Bacskai B.J. (2008). Abeta plaques lead to aberrant regulation of calcium homeostasis in vivo resulting in structural and functional disruption of neuronal networks. Neuron.

[33] Coon A.L., Wallace D.R., Mactutus C.F., Booze R.M. (1999). L-type calcium channels in the hippocampus and cerebellum of Alzheimer’s disease brain tissue. Neurobiol. Aging.

[34] Campbell L.W., Hao S.Y., Thibault O., Blalock E.M., Landfield P.W. (1996). Aging changes in voltage-gated calcium currents in hippocampal CA1 neurons. J. Neurosci..

[35] Choi J., Chandrasekaran K., Demarest T.G., Kristian T., Xu S., Vijaykumar K., Dsouza K.G., Qi N.R., Yarowsky P.J., Gallipoli R. (2014). Brain diabetic neurodegeneration segregates with low intrinsic aerobic capacity. Ann. Clin. Transl. Neurol..

[36] Vogel-Ciernia A., Wood M.A. (2014). Examining object location and object recognition memory in mice. Curr. Protoc. Neurosci..

[37] Haettig J., Stefanko D.P., Multani M.L., Figueroa D.X., McQuown S.C., Wood M.A. (2011). HDAC inhibition modulates hippocampus-dependent long-term memory for object location in a CBP-dependent manner. Learn. Mem..

[38] Mehta B., Snellman J., Chen S., Li W., Zenisek D. (2013). Synaptic ribbons influence the size and frequency of miniature-like evoked postsynaptic currents. Neuron.

[39] Gosrani S.P., Jester H.M., Zhou X., Ryazanov A.G., Ma T. (2020). Repression of eEF2 kinase improves deficits in novel object recognition memory in aged mice. Neurobiol. Aging.

[40] Beckelman B.C., Yang W., Kasica N.P., Zimmermann H.R., Zhou X., Keene C.D., Ryazanov A.G., Ma T. (2019). Genetic reduction of eEF2 kinase alleviates pathophysiology in Alzheimer’s disease model mice. J. Clin. Investig..

[41] Wang J.L., Wang Y., Sun W., Yu Y., Wei N., Du R., Yang Y., Liang T., Wang X.L., Ou C.H. (2021). Spinophilin modulates pain through suppressing dendritic spine morphogenesis via negative control of Rac1-ERK signaling in rat spinal dorsal horn. Neurobiol. Dis..

[42] Morris C.W., Watkins D.S., Shah N.R., Pennington T., Hens B., Qi G., Doud E.H., Mosley A.L., Atwood B.K., Baucum A.J. (2023). Spinophilin Limits Metabotropic Glutamate Receptor 5 Scaffolding to the Postsynaptic Density and Cell Type Specifically Mediates Excessive Grooming. Biol. Psychiatry.

[43] Hongpaisan J., Sun M.K., Alkon D.L. (2011). PKC epsilon activation prevents synaptic loss, Abeta elevation, and cognitive deficits in Alzheimer’s disease transgenic mice. J. Neurosci..

[44] Akram A., Christoffel D., Rocher A.B., Bouras C., Kovari E., Perl D.P., Morrison J.H., Herrmann F.R., Haroutunian V., Giannakopoulos P. (2008). Stereologic estimates of total spinophilin-immunoreactive spine number in area 9 and the CA1 field: Relationship with the progression of Alzheimer’s disease. Neurobiol. Aging.

[45] Gao J., Hu X.D., Yang H., Xia H. (2018). Distinct Roles of Protein Phosphatase 1 Bound on Neurabin and Spinophilin and Its Regulation in AMPA Receptor Trafficking and LTD Induction. Mol. Neurobiol..

[46] Lam M., Chen C.Y., Li Z., Martin A.R., Bryois J., Ma X., Gaspar H., Ikeda M., Benyamin B., Brown B.C. (2019). Comparative genetic architectures of schizophrenia in East Asian and European populations. Nat. Genet..

[47] Liu Z., Peng C., Zhuang Y., Chen Y., Behnisch T. (2020). Direct Medial Entorhinal Cortex Input to Hippocampal CA3 Is Crucial for eEF2K Inhibitor-Induced Neuronal Oscillations in the Mouse Hippocampus. Front. Cell. Neurosci..

[48] Li D., Jing D., Liu Z., Chen Y., Huang F., Behnisch T. (2019). Enhanced Expression of Secreted alpha-Klotho in the Hippocampus Alters Nesting Behavior and Memory Formation in Mice. Front. Cell. Neurosci..

[49] Cetin A., Komai S., Eliava M., Seeburg P.H., Osten P. (2006). Stereotaxic gene delivery in the rodent brain. Nat. Protoc..

[50] Lu Q., Murakami C., Murakami Y., Hoshino F., Asami M., Usuki T., Sakai H., Sakane F. (2020). 1-Stearoyl-2-docosahexaenoyl-phosphatidic acid interacts with and activates Praja-1, the E3 ubiquitin ligase acting on the serotonin transporter in the brain. FEBS Lett..

[51] Wang C., Pan Y., Zhang W., Chen Y., Li C., Zhao F., Behnisch T. (2021). Positive Regulatory Domain I-binding Factor 1 Mediates Peripheral Nerve Injury-induced Nociception in Mice by Repressing Kv4.3 Channel Expression. Anesthesiology.

[52] Yun D., Zhuang Y., Kreutz M.R., Behnisch T. (2018). The role of 19S proteasome associated deubiquitinases in activity-dependent hippocampal synaptic plasticity. Neuropharmacology.

[53] Weng W., Li D., Peng C., Behnisch T. (2018). Recording Synaptic Plasticity in Acute Hippocampal Slices Maintained in a Small-volume Recycling-, Perfusion-, and Submersion-type Chamber System. J. Vis. Exp..

[54] Jing D., Li D., Peng C., Chen Y., Behnisch T. (2019). Role of microtubules in late-associative plasticity of hippocampal Schaffer collateral-CA1 synapses in mice. Neurobiol. Learn. Mem..

[55] Ting J.T., Daigle T.L., Chen Q., Feng G. (2014). Acute brain slice methods for adult and aging animals: Application of targeted patch clamp analysis and optogenetics. Methods Mol. Biol..

[56] Mikhaylova M., Vakhitova J.V., Yamidanov R.S., Salimgareeva M., Seredenin S.B., Behnisch T. (2007). The effects of ladasten on dopaminergic neurotransmission and hippocampal synaptic plasticity in rats. Neuropharmacology.

[57] Behnisch T., Wilsch V.W., Balschun D., Reymann K.G. (1998). The role of group II metabotropic glutamate receptors in hippocampal CA1 long-term potentiation in vitro. Eur. J. Pharmacol..

[58] Shi H., Yu Y., Lin D., Zheng P., Zhang P., Hu M., Wang Q., Pan W., Yang X., Hu T. (2020). beta-glucan attenuates cognitive impairment via the gut-brain axis in diet-induced obese mice. Microbiome.

[59] Walsh R.N., Cummins R.A. (1976). The Open-Field Test: A critical review. Psychol. Bull..

[60] Joca L., Zuloaga D.G., Raber J., Siegel J.A. (2014). Long-term effects of early adolescent methamphetamine exposure on depression-like behavior and the hypothalamic vasopressin system in mice. Dev. Neurosci..

[61] Nogueira Neto J.D., de Almeida A.A., da Silva Oliveira J., Dos Santos P.S., de Sousa D.P., de Freitas R.M. (2013). Antioxidant effects of nerolidol in mice hippocampus after open field test. Neurochem. Res..

[62] Hong X., Liu J., Zhu G., Zhuang Y., Suo H., Wang P., Huang D., Xu J., Huang Y., Yu M. (2014). Parkin overexpression ameliorates hippocampal long-term potentiation and beta-amyloid load in an Alzheimer’s disease mouse model. Hum. Mol. Genet..

[63] Clement Y., Calatayud F., Belzung C. (2002). Genetic basis of anxiety-like behaviour: A critical review. Brain Res. Bull..

[64] Hughes R.N. (2004). The value of spontaneous alternation behavior (SAB) as a test of retention in pharmacological investigations of memory. Neurosci. Biobehav. Rev..

[65] Wang Z.J., Zhao F., Wang C.F., Zhang X.M., Xiao Y., Zhou F., Wu M.N., Zhang J., Qi J.S., Yang W. (2019). Xestospongin C, a Reversible IP3 Receptor Antagonist, Alleviates the Cognitive and Pathological Impairments in APP/PS1 Mice of Alzheimer’s Disease. J. Alzheimers Dis..

[66] Yu X.D., Li A., Li X.Y., Zhou Y., Li X., He Z., Wang L., Reilly J., Tan Z., Xiao Z.Y. (2022). Trans-urocanic acid facilitates spatial memory, implications for Alzheimer’s disease. Physiol. Behav..

[67] Wiseman F.K., Pulford L.J., Barkus C., Liao F., Portelius E., Webb R., Chavez-Gutierrez L., Cleverley K., Noy S., Sheppard O. (2018). Trisomy of human chromosome 21 enhances amyloid-beta deposition independently of an extra copy of APP. Brain.

[68] Leger M., Quiedeville A., Bouet V., Haelewyn B., Boulouard M., Schumann-Bard P., Freret T. (2013). Object recognition test in mice. Nat. Protoc..

[69] Binder S., Baier P.C., Molle M., Inostroza M., Born J., Marshall L. (2012). Sleep enhances memory consolidation in the hippocampus-dependent object-place recognition task in rats. Neurobiol. Learn. Mem..

[70] Raza S.A., Albrecht A., Caliskan G., Muller B., Demiray Y.E., Ludewig S., Meis S., Faber N., Hartig R., Schraven B. (2017). HIPP neurons in the dentate gyrus mediate the cholinergic modulation of background context memory salience. Nat. Commun..

[71] Han C.J., O’Tuathaigh C.M., van Trigt L., Quinn J.J., Fanselow M.S., Mongeau R., Koch C., Anderson D.J. (2003). Trace but not delay fear conditioning requires attention and the anterior cingulate cortex. Proc. Natl. Acad. Sci. USA..

[72] Burman M.A., Simmons C.A., Hughes M., Lei L. (2014). Developing and validating trace fear conditioning protocols in C57BL/6 mice. J. Neurosci. Methods.

