# Supplementary material for: The E3 Ubiquitin Ligase PRAJA1: A Key Regulator of Synaptic Dynamics and Memory Processes with Implications for Alzheimer’s Disease
Source: Int J Mol Sci. 2025 Mar 23;26(7):2909. doi: 10.3390/ijms26072909 (PMC11988436; doi:10.3390/ijms26072909)
Supplement: Supplementary file 1 [file ijms-26-02909-s001.zip › ijms-3487495-supplementary.pdf]

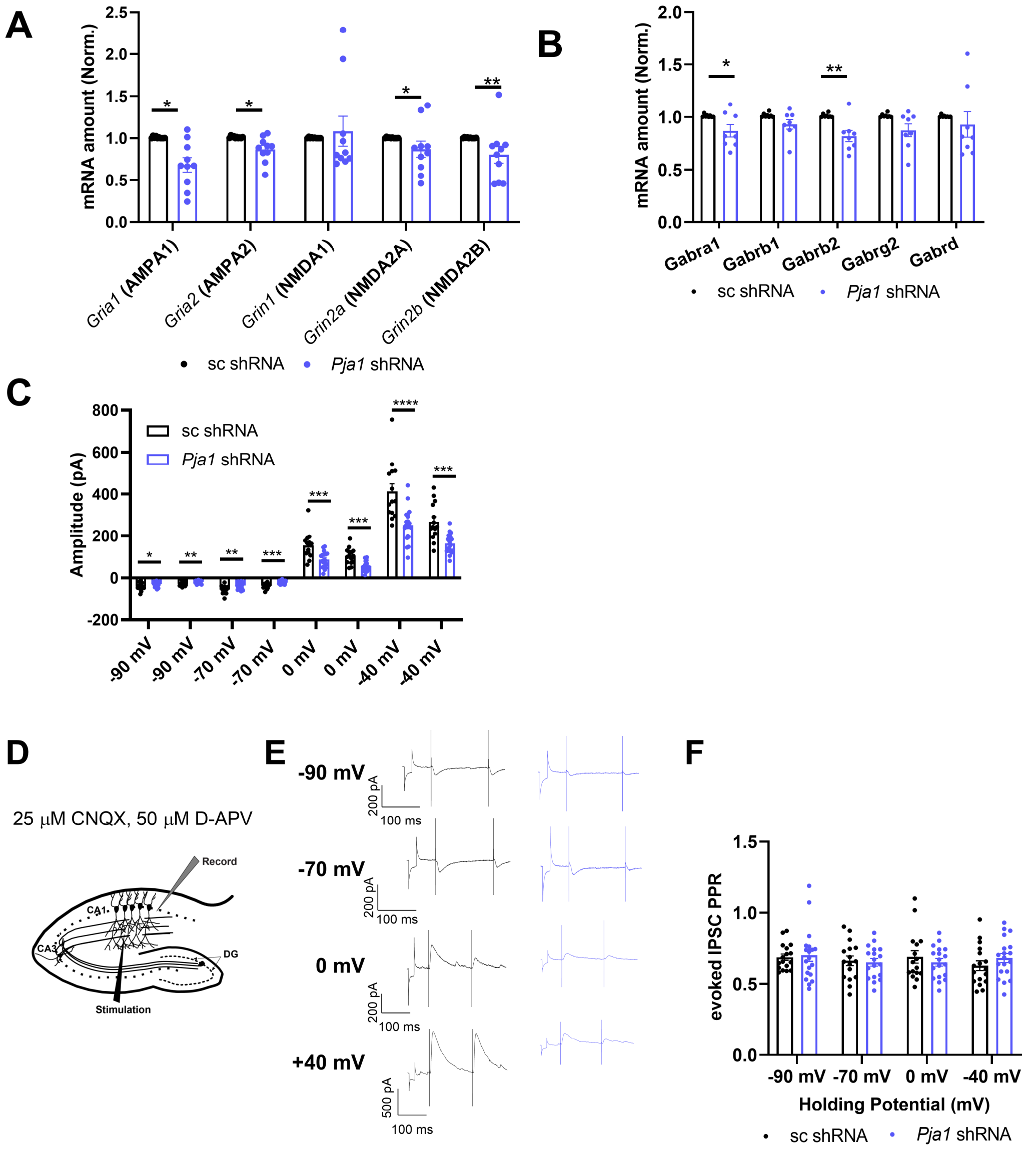

Supplementary Figure S1. Impact of *Pja1* shRNA on mRNA expression of glutamate and GABAA receptor subunits, eIPSC amplitudes, and paired-pulse ratios in the CA1 region of the mouse brain.

(A) mRNA expression of the glutamate receptor Subunits: Relative mRNA levels of *Gria1*, *Gria2*, *Grin2a*, and *Grin2b* in the CA1 region of mice injected with sc shRNA or *Pja1* shRNA. n = 10 per group for each gene. **P* <0.05, ***P* <0.01 (Mann-Whitney test). The data in the graphs are presented as the means ± SEMs and individual data points.

(B) mRNA expression of GABA$_A$ receptor subunits: Relative mRNA expression levels of *Gabra1*, *Gabrb1*, *Gabrb2*, *Gabrg2*, and *Gabrd* in the CA1 region of mice injected with sc shRNA or *Pja1* shRNA. Data are presented as mean ± SEM. n = 8 per group. **P* <0.05, ***P* <0.01 (Mann-Whitney test).

(C) eIPSC amplitude: Summary bar graphs showing eIPSC amplitude at different holding potentials. Data are presented as mean ± SEM. n = 14 cells for sc shRNA, n = 18 cells for *Pja1* shRNA (Mann-Whitney test).

(D) Evoked IPSC (eIPSC) recordings: Schematic diagram illustrating the recording configuration for eIPSCs in the presence of CNQX and D-APV to block excitatory synaptic transmission. DG: dentate gyrus.

(E) Representative eIPSC recordings: Representative traces of eIPSC recorded from CA1 pyramidal neurons at a holding potential of −90 mV, -70 mV, 0 mV, +40 mV, sc shRNA: black; *Pja1* shRNA: blue.

(F) eIPSC Paired-Pulse Ratio: Summary bar graphs showing eIPSC paired-pulse ratio (PPR) at different holding potentials. Data are presented as mean ± SEM. n = 14 cells for the sc shRNA group, n = 18 cells for the *Pja1* shRNA group.

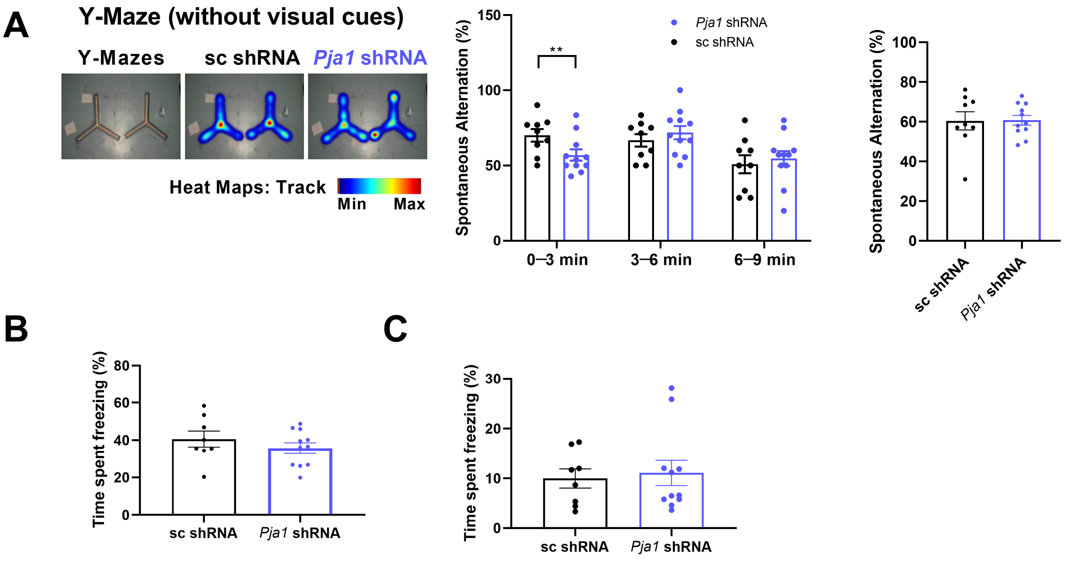

Supplementary Figure S2. Effects of Pja1 shRNA on spontaneous alternation ratios in the Y-maze and freezing behavior in context and tone fear tests.

(A) Y-maze test: *Left:* Schematic diagram of the Y-maze. *center*: Summary data for spontaneous alternation ratio in the Y-maze without visual cues across different time periods. 0-3min: *Pja1* shRNA: 56.90 ± 3.759%, sc shRNA: 70.00 ± 4.195%, *P* <0.05, Mann-Whitney test. *Right:* Summary data for spontaneous alternation ratio in the Y-maze without visual cues in 10min. n = 9 for the sc shRNA group, and n = 11 for the *Pja1* shRNA group. Data are presented as mean ± SEM. *P* >0.05, ***P* <0.01 (Mann–Whitney test).

(B) Percentage of freezing time during the context fear test, and **(C)** percentage of freezing time during the tone fear test. The data are presented as the means ± SEMs of individual data points. *P* >0.05, n = 9 for the sc shRNA group, and n = 11 for the *Pja1* shRNA group (Mann-Whitney test).

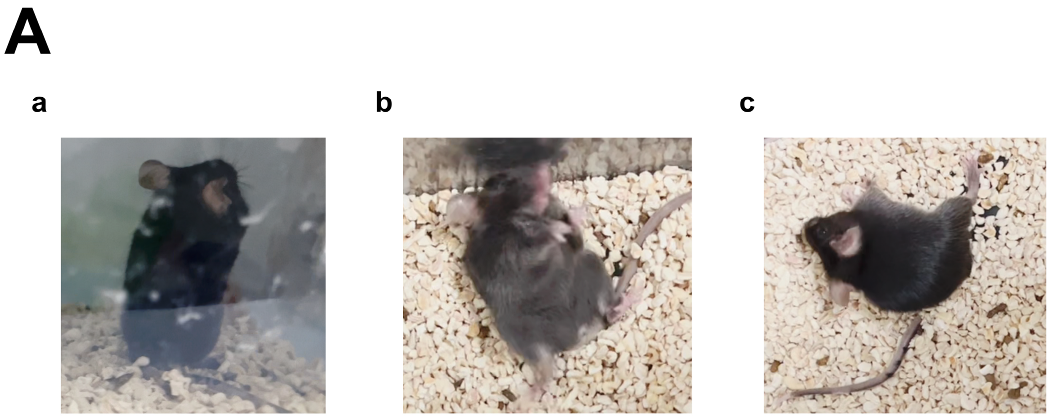

Supplementary Figure S3. Seizure-like behavioral symptoms in Pja1 overexpressing mice. **(A) *Pja1*-OE mice presented seizure-like behavioral symptoms.** Snapshot images showing seizure-like behavioral symptoms, such as (a) rigidity, (b) muscle twitching and (c) falling, in *Pja1*-OE mice.

Supplementary Table S1. Object Recognition Test Data: Exploration Times and Discrimination Index.

Number.	Exploration time (s)	Novel object explores time (s)	Familiar object explores time (s)	20-second exploration criterion	DI (%)
OC-1	180	7.07	13.2	√	−30.24173656
OC-2	148	12.73	7.44	√	26.22706991
OC-3	180	11.72	9.26	√	11.72545281
OC-4	236	13.2	8.25	√	23.07692308
OC-5	470	13.32	9	√	19.35483871
OC-6	300	11.62	8.57	√	15.10648836
OC-7	190	11.47	8.56	√	14.52820769
OC-8	185	15.11	5.61	√	45.84942085
OC-9	360	12.34	8.4	√	18.99710704
OC-10	220	18.34	3.37	√	68.95439889
OE-1	120	12.52	10.58	√	8.398268398
OE-2	210	11.34	10.12	√	5.68499534
OE-3	>600	/	/	×	/
OE-4	>600	/	/	×	−25.84704743
OE-5	>600	/	/	×	/
OE-6	67	20.50	0	√	100
OE-7	>600	/	/	×	/
OE-8	360	11.06	9.01	√	10.21425012
OE-9	110	12.25	11.72	√	2.211097205
OE-10	140	10.15	11.57	√	−6.537753223

Object recognition memory results: Exploration Time: Time taken to reach the 20-second exploration criterion during the testing phases of ORM. DI: Discrimination index. Novel object explores time (s): the time of mice spent to explore the novel object. Familiar object explores time (s): the time of mice spent to explore the familiar object. n = 10 for each group.

Supplementary Table S2. Object Location Test Data: Exploration Times and Discrimination Index.

Number.	Total time (s)	Novel location explores time (s)	Familiar location explores time (s)	20-second exploration criterion	DI (%)
OC-1	500	12.72	8.59	√	19.38
OC-2	204	16.64	7.84	√	35.94
OC-3	150	17.08	3.19	√	68.52
OC-4	290	12.56	10.15	√	10.61
OC-5	440	14.93	5.48	√	46.30

OC-6	400	18.17	5.51	√	53.46
OC-7	240	15	5.75	√	44.57
OC-8	290	6.83	16.9	√	−42.43
OC-9	314	16.3	6.27	√	44.43
OC-10	264	14.92	5.84	√	43.73
OE-1	190	17.12	4.1	√	61.35
OE-2	378	19.44	1.03	√	89.93
OE-3	>600	/	/	×	/
OE-4	>600	/	/	×	/
OE-5	>600	/	/	×	/
OE-6	140	19.91	1.99	√	81.82
OE-7	>600	/	/	×	/
OE-8	>600	/	/	×	/
OE-9	118	19.77	3.77	√	67.96
OE-10	154	13.61	8.36	√	23.89

Object location tests data table: Exploration Time: Time taken to reach the 20-second exploration criterion during the testing phases of OLM. DI: Discrimination index. Novel location explores time: the time of mice spent to explore the novel location. Familiar location explores time: the time of mice spent to explore the familiar object (n = 10 for each group).
